# Protocol Study for the Evaluation of Non-Opioid Balanced General Anaesthesia in Cardiac Surgery with Cardiopulmonary Bypass: A Randomised, Controlled, Multicentric Superiority Trial (OFACAR Study)

**DOI:** 10.3390/jcm12175473

**Published:** 2023-08-23

**Authors:** Pierre-Grégoire Guinot, Guillaume Besch, Bastien Hameury, Tommy Grelet, Paul Michel Mertes, Maxime Nguyen, Belaid Bouhemad, on behalf of the OFACAR Study Group

**Affiliations:** 1 Department of Anaesthesiology and Critical Care Medicine, Dijon University Medical Centre, 21000 Dijon, France; bastien.hameury@gmail.com (B.H.); maxime.nguyensoenen@gmail.com (M.N.); belaid.bouhemad@chu-dijon.fr (B.B.); 2 University of Burgundy and Franche-Comté, LNC UMR1231, F-21000 Dijon, France; 3 Department of Anaesthesiology and Critical Care Medicine, Besançon Regional University Medical Centre, 25030 Besançon, France; gbesch@chu-besancon.fr (G.B.); tgrelet@chu-besancon.fr (T.G.); 4 EA3920, University of Franche-Comté, 25000 Besançon, France; 5 Department of Anesthesia and Critical Care, University Hospital of Strasbourg, 67200 Strasbourg, France; paul-michel.mertes@chru-strasbourg.fr

**Keywords:** opioid-free anaesthesia, lidocaine, opioid, general anaesthesia, outcomes, acute kidney injury, sepsis

## Abstract

Opioid-free anaesthesia (OFA) is general anaesthesia based on the use of several non-opioid molecules that aim to have an analgesic effect, decrease the sympathetic response, decrease hormonal stress, and decrease the inflammatory response during surgery. Although this approach to anaesthesia is regularly used in clinical practice, it remains a novel approach. The literature on this anaesthesia modality finds a number of positive effects on cardiac, respiratory, and cognitive function but no randomised study evaluated these effects during cardiac surgery where there is a high incidence of postoperative complications. The main aim of the study is to compare OFA vs. standard balanced opioid general anaesthesia on the incidence of postoperative complications and the length of stay in intensive care and hospital. OFACAR is a multicentric, randomised, controlled, superiority, single-blind, two parallel-arm clinical trial in patients undergoing cardiac surgery with cardiopulmonary bypass. We compared a balanced general anaesthesia without opioids (OFA group) vs. a balanced opioid general anaesthesia with sufentanil (control group). One hundred and sixty patients will be enrolled in each treatment group. The primary endpoint is a composite one which corresponds to the occurrence of at least one of the postoperative complications, defined according to European standards within 30 days after surgery. Recruitment started in September 2019, and data collection is expected to end in November 2024.

## 1. Introduction

Intraoperative administration of opioids, in combination with hypnotics and muscle relaxants, is considered a key component of balanced general anaesthesia [1]. However, the use of opioids is associated with several side effects (hypoxaemia, confusion, postoperative pain, postoperative nausea, and vomiting), which may be responsible for an increase in operative morbidity and may be a source of postoperative misuse and addiction [2]. 

Opioid-free anaesthesia (OFA) is general multimodal anaesthesia based on the use of hypnotics and non-opioid analgesics (lidocaine, ketamine, corticoid, and esmolol). OFA has been used in the past 10 years with several positive clinical effects in randomised and non-randomised studies [3,4,5,6]. The effects are better analgesia with reduced postoperative morphine consumption, better lung function, better hemodynamic stability and better postoperative cognitive function [4,6,7]. To date, no randomised trial has evaluated OFA in cardiac surgery with cardiopulmonary bypass (CPB). Only observational studies have suggested an improvement in analgesia and a decrease in some postoperative complications [6,7]. Cardiac surgery is a major surgery that most often includes an elderly population, with a high incidence of postoperative complications (up to 50%) [8]. The occurrence of these complications is the result of a complex interaction between the patient’s preoperative condition, anaesthesia, surgery and CPB. It has also been demonstrated that each molecule used in OFA protocols can individually decrease the inflammatory response to CPB and respiratory complications and improve postoperative cognitive function and operative haemodynamic stability. In addition, the absence of opioid use would avoid the side effects associated with their use. 

Thus, the hypothesis of the present study is that OFA during cardiac surgery with CPB is associated with an improvement of intraoperative haemodynamic stability, a reduction in the incidence of postoperative complications, thus a reduction in the length of stay in intensive care and hospital. The main objective of the study is to demonstrate that OFA decreases a composite score of cardiovascular complications.

## 2. Material and Methods

### 2.1. Study Design

This study is a randomised, controlled, superiority, single-blind, two parallel-arm, multicentric clinical trial in patients undergoing cardiac surgery with CPB. We will compare a balanced general anaesthesia without morphine vs. a standard balanced general anaesthesia (with opioids). This study is conducted at the tertiary university hospitals of Dijon, Besançon and Strasbourg, in France.

Only the anaesthetist and the anaesthetist nurse in charge of the patient during the surgery are aware of the allocated treatment. They are not involved in the management of the patients before and after the surgery. Neither the surgeons, the nurse, the intensivist, nor the cardiologist are informed of the allocation group to maintain blindness during their time in the ICU and the hospital stay following surgery. The patient is kept blind about the randomisation group throughout their stay. 

Information about the study protocol is given during the anaesthesia consultation by one of the investigators (between 48 h and 2 months before the cardiac surgery), and all patients give their written informed consent during the preoperative visit (baseline visit) the day before the surgery. Inclusion and non-inclusion criteria are checked before the final inclusion and randomisation. 

### 2.2. Ethics Statement 

This study was approved by an independent ethics committee (French CPP Sud Est VI), received the authorisation of the Agence Nationale de Sécurité du Médicament et des Produits de Santé and was registered on the Clinical Trial Registration (n° 2021-000066-16, NCT04886453) before the inclusion of the first patient. The first patient was included in August 2021, and the last patient follow-up is planned for May 2024.

### 2.3. Study Population

We included adult patients from Dijon University Hospital, Strasbourg University Hospital and Besançon University Hospital undergoing cardiac surgery with CPB. The inclusion and non-inclusion criteria are provided in Table 1.

### 2.4. Randomisation 

Participants are randomised to receive either opioid-free anaesthesia (interventional group: OFA) or conventional anaesthesia with sufentanil (control group). This allocation is stratified by the centre and Euroscore 2. The 1:1 ratio randomisation is performed by an investigator not in charge of the patient on the day of the cardiac surgery to avoid bias, using the CleanWeb^TM^ software (V 176.1.0, Telemedicine Technologie SAS, Boulogne Billancourt, France), and this process is controlled by an identification using a password. The compliance of the data with the inclusion and non-inclusion criteria is checked a second time at this point. The treatment allocation algorithm of the randomisation was established by the Research Methodology Support Unit (USMR) of the Dijon Bourgogne University Hospital, which also offered technical support and keeps a confidential record of the full randomisation process.

### 2.5. Study Intervention 

Institutions follow international guidelines for the maintenance and withdrawal of preoperative medications [9,10]. Anaesthesia, CPB, and postoperative management are standardised for all patients and follow usual guidelines [10,11,12]. During the surgery, only the anaesthesia protocol differs. 

#### 2.5.1. OFA Group 

At induction, all patients receive intravenous ketamine (0.5 mg kg^−1^), dexamethasone (0.1 mg kg^−1^), lidocaine (1.5 mg kg^−1^), and magnesium sulphate (30 mg kg^−1^). Anaesthesia is maintained with the Schnider model target-controlled infusions of propofol (started at an effect site concentration of 2–4 mcg mL^−1^) and continuous infusion of lidocaine (1.5 mg kg^−1^ h^−1^ until sternotomy, then 1 mg kg^−1^ h^−1^ until end of surgery). Sedation is titrated by using the bispectral index (Covidien, Boulder, CO, USA), aiming for a value between 40 and 60.

#### 2.5.2. Control Group 

In the control group, anaesthesia is induced and maintained with sufentanil with an effect site concentration started at 0.5 ng mL^−1^ and propofol with an effect site concentration started at 2–4 ng mL^−1^. Then, anaesthesia is maintained with sufentanil by using the Gepts target-controlled infusion model and propofol by using the Schnider model target-controlled infusions (started at an effect site concentration of 2–4 mcg mL^−1^). Sufentanil is stopped at the end of the procedure. Sedation is titrated based on the bispectral index (Covidien, Boulder, CO, USA), aiming for a value between 40 and 60.

For both groups, tracheal intubation is facilitated via the intravenous use of cisatracurium (0.15 mg kg^−1^) or atracurium (0.4 mg kg^−1^). Arterial hypertension (systolic arterial pressure > 140 mmHg) is treated with esmolol in case of tachycardia (heart rate > 80 bpm) or urapidil/nicardipine in case of heart rate < 80 bpm. Fluid therapy is optimised during and after the surgery according to French guidelines [12,13]. Fluid therapy optimisation is based on Stroke Volume (SV) optimisation.

Ventilation strategy consists of a lung protective strategy (tidal volume of 6 to 8 mL kg^−1^ ideal body weight, positive end-expiratory pressure of 5 to 14 cm H_2_O, and recruitment manoeuvres) before and after CPB, and in the ICU; FiO_2_ set to obtain SpO_2_ between 95 and 99%. 

#### 2.5.3. ICU Management

At the end of surgery all patients are sedated with propofol infusion, and the lungs are mechanically ventilated until haemodynamic stability and normothermia are obtained, and blood loss is considered acceptable (less than 1 mL kg^−1^ h^−1^) [14]. A specialized team (trained in the care of postoperative cardiac surgery) manage all patients. Circulatory support is guided by using institutional protocols to achieve predefined endpoints: mean arterial pressure > 65 mmHg, cardiac index > 2.2 L min^−1^ m^−2^, and urine output > 0.5 mL kg^−1^ h^−1^. Electrocardiogram, pulse oxygen saturation, and central venous blood pressure are continuously monitored. Scheduled blood tests include arterial/venous blood gas measurements on admission to the ICU and then several times a day on the request of the attending physician.

Analgesia is standardised and comprises paracetamol (15 mg kg^−1^ every 6 h) use and morphine. Morphine is initially administered intravenously via a titration of 2–3 mg to obtain a visual analogue pain scale (VAS < 3) and then via the use of a patient-controlled morphine pump. Complementary analgesia is allowed when analgesia is insufficient (VAS > 3) despite treatment with morphine (20 mg for titration or for the cumulated dose over the last 4 h) and paracetamol. Complementary analgesia comprises ketoprofen (up to 200 mg per day), and/or tramadol, and/or nefopam. Pain is assessed every 4 h. The use of locoregional analgesia (epidural analgesia, paravertebral block, serratus block, intercostal block, and interpectoral block) is prohibited.

### 2.6. Data Collection 

The data are prospectively collected in an electronic CRF (e-CRF) created with CleanWeb software by a data manager. Demographic and surgical data are collected at the baseline visit. All patient-related data are anonymously prospectively noted in the e-CRF by a clinical research associate who is blind to the group assignment. Data consists of: gender, age, weight; size; ASA score; Euroscore 2; Apfel score; comorbidities; ventricular aneurysm; preoperative left ventricular ejection fraction (%); preoperative creatinine levels; drug consumption and the type of surgery; episodes of hypertension; hypotension; bradycardia and tachycardia; CPB time; aortic clamping time; duration of anaesthesia; biological assessment at the end of the bypass surgery; electrosystolic entrainment; total dose of propofol; sufentanil; lidocaine; ketamine; heparin; and protamine; total dose of insulin; total fluid balance; transfusion of red blood cells; tranexamic acid; catecholamine use (dose, type, duration); operative diuresis; veno-arterial ECMO; and intra-aortic counter pulsation balloon. Postoperative data comprise: time to extubation; complications; SOFA score; arterial lactates; catecholamine (type, dose, duration); total dose of insulin; total dose of morphine; postoperative pain episodes at rest and cough (VAS > 3) and the number of episodes of nausea–vomiting following 48 h after extubation; standard biological; additional analgesia. On discharge from the ICU, a QoR15 questionnaire is performed by a nurse who is blind to group assignment. 

**Figure 1:**
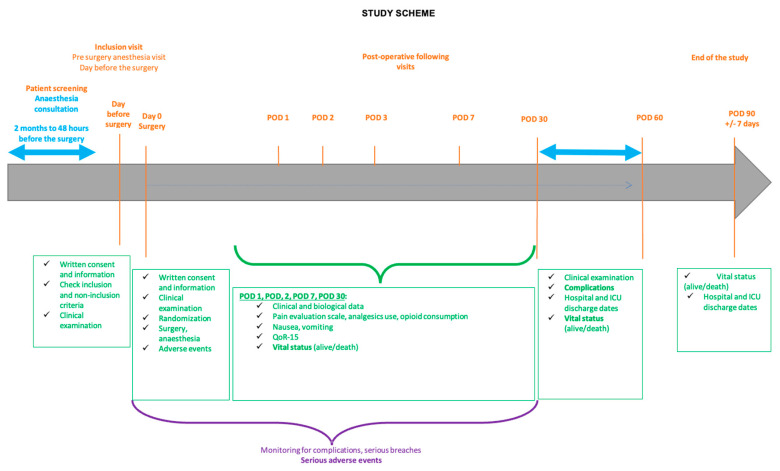
Scheme of the OFACAR study.

Complications are prospectively noted from the day of operation until the 60th postoperative day (POD 60). A post-surgical consultation is performed at POD 30 and POD 60. This consultation could be replaced by a telephone call at POD 60 if it does not take place, but the information collected must correspond to the patient’s condition at POD 30. A telephone call is made at POD 90 to collect the vital status. The study scheme is presented in Figure 1.

### 2.7. Objectives 

The main objective is to demonstrate the superiority of balanced general anaesthesia without opioids (OFA) over a balanced opioid anaesthesia (with sufentanil ) on the occurrence of at least one of the serious postoperative complications at day 30 following cardiac surgery with CPB. 

The secondary objectives are to compare the two strategies in terms of the occurrence of each type of serious complication within the first 30 days after surgery, morphine consumption within 48 h of extubation, the quality of postoperative analgesia during the first 48 h after surgery, the quality of post-surgical recovery at discharge from the intensive care unit, the occurrence of arrhythmia due to atrial fibrillation and/or atrial flutter, the occurrence of vasoplegic syndrome, the occurrence of postoperative infectious episodes, the time to extubation of the patient in intensive care, the occurrence of non-serious side effects associated with morphine (nausea and vomiting), the postoperative length of stay in the ICU, the total postoperative hospital stay and the mortality at POD 90.

### 2.8. Outcomes 

The primary endpoint is a composite endpoint, which comprises the occurrence of at least one of the postoperative complications, defined according to European standards (6), within 30 days after surgery, as provided in Table 2. The complications are defined according to international recommendations (ESAIC, ESICM) [15,16]. 

The secondary endpoints are the occurrence of each type of serious complication within the first 30 postoperative days: total cumulative dose of morphine (in milligrams), the number of episodes of postoperative pain (VAS > 3) at rest and on coughing within 48 h of tracheal extubation, the post-surgical recovery by questionnaire (QoR15), the number of patients with an atrial rhythm disorder (arrhythmia due to atrial fibrillation or atrial flutter), the number of patients with an infectious episode (pneumonia, urinary tract infection, surgical site infection, or bacteraemia), the time to extubation of the resuscitation patient (hours), the number of episodes of postoperative nausea and vomiting in the first 48 h after extubation, the ICU stays in days, and the hospital stay in days.

For patients included at the hospital of Dijon, an additional blood sample specimen is constituted at different times: before the surgery, at the end of the CPB, 4 h after the CPB, on the first POD, and on the second POD.

### 2.9. Statistics 

#### 2.9.1. Sample Size Calculation 

Assuming a frequency of complications in the standard group of 60% and a decrease of 20% in the OFA group (minimal expected difference given the simultaneous consideration of several clinical events), 144 patients per group are required, i.e., a total of 288 patients with a power of 90% and an alpha risk (two-sided) of 5%. Considering the composite nature of the primary endpoint, we chose a power of 90%. Anticipating 10% non-evaluable data for the primary endpoint, we fixed the sample size to 320 patients, 160 in the OFA group and 160 in the control group (Supplementary Materials, consort Flow chart diagram). Sample size calculations were based on previously published studies on OFA in cardiac surgery and institutional databases [6,7].

#### 2.9.2. Data Analysis 

The main analysis will be conducted on an intention-to-treat basis and completed by a per-protocol (Supplementary Materials; flow chart diagram). The main conclusion of the trial will be on the intention-to-treat analysis only. The quantitative variables will be described by the mean ± standard deviation and the median [25–75% IQR] and qualitative variables will be described by their number (%). The characteristics of the two groups will be compared using the usual univariate test: Chi-square test or Fisher’s exact test for qualitative variables and Student’s or Wilcoxon’s test for quantitative variables, depending on the test conditions. 

Logistic regression adjusted on the stratification factors (centre and Euroscore 2) will be performed to analyse the effect of the type of anaesthesia on the rate of occurrence of postoperative complications.

The analysis of secondary endpoints will be performed using an ANOVA adjusted for stratification factors or the Wilcoxon test depending on the distribution of the variables for quantitative variables and a logistic regression adjusted for stratification factors for categorical variables. The time to extubation will be described and compared between the two groups using Kaplan–Meier curves and a log-rank test if the application conditions are met. If factors at inclusion appear unbalanced between the two groups, multivariate analyses will be performed including these factors in addition to the stratification factors. 

An intermediate analysis will be performed after the inclusion of 25% and 50% of patients. Thus, the safety of the trial (rate of serious adverse events between inclusion and POD 30), as well as the rate of occurrence of postoperative complications, will be described and analysed according to the treatment arm. These results will be submitted to the independent safety monitoring board, who will decide whether to stop the trial. The final statistical analyses will be performed using a significant level of 5%.

The intermediate analyses will use an alpha risk of 0.001 for each safety analysis of the trial, according to the method proposed by Peto–Haybittle, in order not to modify the significance level of the final main analysis. The statistical analysis is performed using SAS Software (version 9.4). 

#### 2.9.3. Ancillary Study 

An ancillary study that will mix the data (meta data analysis) of the present study and a second multicentric RCT that compare OFA using dexmedetomidine and opioid general balanced anaesthesia in cardiac surgery is planned.

## 3. Discussion 

The expected results of the present study will (or will not) confirm the clinical benefit of avoiding intra-operative use of opioids during cardiac surgery by decreasing postoperative complications in relation to better hemodynamic management and decreasing morphine-related side effects.

This protocol is part of a global approach to improve peri-operative care, such as an assisted recovery programme after surgery [19]. Cardiac surgery remains a high-risk surgery for which we need to improve our practices as the incidence of complications, length of hospital stays and costs associated with this surgery are high. The reduction in peri-operative complications, length of stay in ICU and hospital stays would also be cost and human saving, and therefore, permit a public health benefit. OFA is based on the use of multimodal non-opioid medications (lidocaine, corticoid, ketamine, and magnesium) that decrease the sympathetic response (beta-blocker and lidocaine), decrease the hormonal stress (beta-blocker, lidocaine, and corticoid), and decrease the inflammatory response (lidocaine, dexamethasone, ketamine, and beta-blocker) to surgery and CPB [20,21]. Although this approach is regularly used in clinical practice, it remains a novel approach in cardiac surgery. The literature on OFA demonstrates a peri-operative safety profile; OFA is not associated with more deleterious haemodynamic events [7]. Avoidance of opioids may also be associated with fewer respiratory side effects, especially in bariatric and cardiac surgery, with a decrease in postoperative apnoea episodes, respiratory failure, use of non-invasive ventilation, and an improvement in oxygen saturation [7]. OFA allows better postoperative alertness: patients seem less confused and more aware. The published protocols of this approach are easily and safely implemented. 

However, OFA is subject to risk in cardiac surgery related to the use of intravenous lidocaine during the anaesthetic period. A significant number of studies have validated the use of lidocaine in cardiac surgery with CPB for periods of up to 48 hours. The dosages proposed in this protocol are associated with serum concentrations below the toxicity thresholds of lidocaine, and the duration of administration is limited to the operating room [22]. Apart from the risks associated with overdosing, no other risks are expected. A risk could be cardiac/rhythmic with the use of dexmedetomidine which we do not use in this protocol [23].

This study will be the first randomised study in cardiac surgery to evaluate the superiority of balanced non-opioid general anaesthesia (OFA) over balanced opioid general anaesthesia. This study follows retrospective studies that have demonstrated these effects with all the bias and limitations of non-randomised retrospective studies [6,7]. This approach is important because it is part of a global approach to optimise the peri-operative management of cardiac surgery patients in order to reduce the incidence of complications (described previously and in the objectives) and the length of hospital stay [24]. The use of morphine is associated with a number of side effects that can increase morbidity and mortality [25]. Amongst these, two aspects are important: disorders related to intraoperative hypoperfusion and postoperative neuro-cognitive disorders, which can affect up to 50% of patients.

A potentially counterintuitive aspect of our OFA protocol pertains to the standardisation of postoperative pain management, achieved through the administration of morphine to all patients. The beneficial outcomes associated with OFA could potentially extend beyond solely avoiding intra-operative opioid use, encompassing the protective effects these interventions might exert on organs [20,21]. In light of this, the utilisation of postoperative morphine might not necessarily compromise the anticipated outcomes. While the inclusion of morphine might initially appear contrary to our OFA objectives, our belief is that this approach ensures consistent patient care and ethical practices while allowing us to investigate the primary interventions performed during surgery in an unbiased manner [10].

In conclusion, the expected study results will lead to an improvement in the management of peri-operative cardiac surgery patients with cardiopulmonary bypass through a decrease in the incidence of major postoperative complications. 

## Figures and Tables

**Table 1:** Inclusion and non-inclusion criteria of the study.

Inclusion criteria	Patients with free, written, and informed consent Patients of legal age Patients requiring cardiac surgery scheduled with CPB and of type: aortic valve surgery, mitral valve surgery, tricuspid valve surgery, atrial myxoma, coronary artery bypass grafting, aortic surgery, or combined surgery	
Non-inclusion criteria	Patients not affiliated with or not benefiting from a social security scheme Patient under legal protection (guardianship, trusteeship) Person subject to a legal protection measure Pregnant, parturient, or breastfeeding women A person who is incapable or not in a position to give consent Patient included once in the study Patient requiring emergency surgery within 24 h Patients with hypersensitivity to local anaesthetics or opiates or to any of the excipients in the products used Patients on antidepressants, neuroleptics such as non-selective (iproniazid), selective A (moclobemide), selective B (selegiline), gabalin (Neurontin^®^), pregabalin	Patient with an atrioventricular conduction disorder without a device Chronic opioid use and/or painkiller use Patient with prolonged QTc (>450 ms) on preoperative EKG Patients with severe hepatic insufficiency (PT < 30%) Patient suffering from respiratory insufficiency (Long-term oxygen therapy patient except for OSA) Patient with unbalanced epilepsy Patient with preoperative cognitive dysfunction (MMS < 24) Patient with intracranial hypertension Patients with chronic renal failure (dialysis, creatinine > 200 µmol L^−1^) Patient with porphyria Patient treated with linezolid (Zyvoxid^®^) Patient with severe arterial hypotension (systolic arterial pressure < 90 mmHg)

**Table 2:** Postoperative complications defined according to European standards.

Postoperative neurological dysfunction	Resuscitation delirium assessed using the Confusion Assessment Method for the Intensive Care Unit (CAM-ICU) [17] Stroke diagnosed on brain computed tomography
Acute renal failure	Increase of at least 50% and/or 26.5 micromol/L in postoperative creatinine from preoperative baseline and/or diuresis less than 0.5 mL/kg/hr on 6 h (KDIGO International Society of Nephrology definition) [15]
Acute respiratory failure	Non-invasive ventilation or optiflow, oro-tracheal intubation for more than 24 hours, or to oro-tracheal re-intubation Acute Respiratory Distress Syndrome, ARDS (defined by Berlin definition)
Cardiovascular complications	Cardiogenic shock defined by treatment with inotropic agents (dobutamine, milrinone, levosimendan, epinephrine) Postoperative myocardial damage (4th universal definition of myocardial infarction) [18] Myocardial infarction (4th universal definition of myocardial infarction) Ventricular arrhythmia requiring treatment (ventricular tachycardia, ventricular fibrillation)
Death at D30	

## Data Availability

Not applicable.

## Supplementary Materials

The following supporting information can be downloaded at: https://www.mdpi.com/article/10.3390/jcm12175473/s1, File S1: Flow chart diagram of the study; File S2: Information Letter and Consent Form given to the participants (French version); File S3: Protocol submitted to independent committee and approved (French version).
