# Supplementary material for: Protocol Study for the Evaluation of Non-Opioid Balanced General Anaesthesia in Cardiac Surgery with Cardiopulmonary Bypass: A Randomised, Controlled, Multicentric Superiority Trial (OFACAR Study)
Source: J Clin Med. 2023 Aug 23;12(17):5473. doi: 10.3390/jcm12175473 (PMC10487869; doi:10.3390/jcm12175473)
Supplement: Supplementary file 1 [file jcm-12-05473-s001.zip › jcm-2479803-supplementary.pdf]

Call for projects: PHRCI 2019
N°RCB : 2021-000066-16
NCT : NCT04886453

EVALUATION OF NON-OPIOID BALANCED GENERAL ANAESTHESIA IN CARDIAC SURGERY WITH cardiopulmonary bypass: A multi centric RANDOMISED CLINICAL TRIAL SUPERIORITY study

OFACAR

Version 3 dated 04/01/2022
Approved on 11/02/2022 by the CPP Sud-Est VI

Version 2 dated 04/10/2021
Approved on 23/11/2021 by the CPP Sud-Est VI
Approved on 18/11/2021 by the ANSM

Version 1.1 of 25/02/2021
Approved on 23/03/2021 by the CPP Sud-Est VI
Approved on 17/03/2021 by the ANSM

Phase III clinical trial

Category 1: Interventional research

PROMOTER	COORDINATING INVESTIGATOR
**Dijon University Hospital Burgundy**<br>14 rue Gaffarel 21079 DIJON<br><br>Tel : 03-80-29-50-13	**Professor BOUHEMAD Belaid**<br>DIJON BOURGOGNE University Hospital<br>Anaesthesia and Intensive Care Department<br>1 Boulevard Jeanne d'Arc<br>21079 DIJON CEDEX

Insurance :

Société Hospitalière d'Assurances Mutuelles (SHAM)
Policy no. 129.234

INVESTIGATORS AND ADMINISTRATIVE STRUCTURE OF THE STUDY

**Promoter**	CHU DIJON BURGUNDY<br>14, rue Gaffarel<br>21079 DIJON CEDEX
**Head of Research**	Mrs Florence MARTEL<br>Clinical Research and Innovation Department<br>1 Bd Jeanne d'Arc<br>21079 DIJON CEDEX<br>03.80.29.50.13
**Coordinating investigator**	Professor Belaid BOUHEMAD<br>DIJON BOURGOGNE University Hospital<br>Anaesthetics and Intensive Care Department<br>1 Boulevard Jeanne d'Arc<br>21079 DIJON CEDEX<br>Tel: 03.80.29.35.28 belaid.bouhemad@chu-dijon.fr
**Methodologist / Statistician**	**Doctor Agnès SOUDRY-FAURE**<br>Dijon University Hospital Burgundy<br>Methodological Research Support Unit, DRCI<br>14 rue Paul Gaffarel - BP 77908<br>21079 DIJON Cedex Tel:<br>03.80.29.56.54<br>agnes.soudry@chu-<br>dijon.fr
**Responsible for vigilance**	**Doctor Camille FLECK**<br>Dijon University Hospital Burgundy<br>Clinical Research and Innovation Delegation<br>DRCI Vigilance Unit<br>14 rue Gaffarel<br>21079 DIJON Cedex<br>Tel : 03.80.29.55.80 - Fax : 03.80.29.36.90<br>Vigilance.ec@chu-dijon.fr
**Investigators<br>(doctors with doctorates)**	List provided in an independent document

Associated Units :

CRB **Centre de Ressources Biologiques Ferdinand Cabanne**

CIC

Other

Please specify:

PROTOCOL AMENDMENT TRACKING TABLE

M S A NUMBER	Protocol version	Changes	Approval date PPC	Approval date ANSM
	V1 of 21/12/2020	Version submitted to the CPP and ANSM		
	V1.1 of 25/02/2021	Version 1 modified in response to the ANSM and the CPP	23/03/2021	17/03/2021
1	V 2 of 04/10/2021	Reorganisation and clarification of non-inclusion criteria<br>Elimination of biological assessment and pain evaluation at H+6<br>Conservation of the collection of biological samples	23/11/2021	18/11/2021
2	V 3 of 04/01/2022	Correction of part<br>PV	11/02/2022	

SYNOPSIS

**Title of the study**	EVALUATION OF NON-OPIOID BALANCED GENERAL ANAESTHESIA IN CARDIAC SURGERY WITH cardiopulmonary bypass: A multi centric RANDOMISED CLINICAL TRIAL SUPERIORITY study (OFACAR)
**Registration**	2021-000066-16
**Developer**	Dijon University Hospital
**Investigators**	Principal : Pr Belaid BOUHEMAD, Department of Anaesthesia and Intensive Care, Dijon Burgundy University Hospital
**Methodologist**	DR AGNES SOUDRY-FAURE
**Rational**	Intraoperative administration of opioids, in combination with hypnotics and muscle relaxants, is considered a key component of anaesthesia. However, the use of opioid is associated with a number of side effects (hypoxaemia, confusion, postoperative pain, postoperative nausea and vomiting) which may be responsible for an increase in operative morbidity and mortality, and may also be a source of misuse and postoperative addiction.<br>Opioid-free anaesthesia (OFA) is a general anaesthetic based on the use of hypnotics and non-opioid analgesics (lidocaine, ketamine, dexamethasone, esmolol). This technique has been used for 10 years and has demonstrated a number of positive effects on cardiac function in randomised and non-randomised studies (1):<br>- improved analgesia and reduced postoperative opioid consumption,<br>- improved respiratory function,<br>- better haemodynamic stability,<br>- better postoperative cognitive function.<br>Currently, a randomised trial in non-cardiac surgery is underway to assess the impact of DAO on immediate postoperative respiratory complications (2). To date, no randomised trial has evaluated the effect of DAO during cardiac surgery. Only one retrospective study from our group found an improvement in analgesia and a reduction in postoperative complications. Cardiac surgery is a major operation which most often involves an elderly population, with a high incidence of postoperative complications (up to 50%). The occurrence of these complications is the result of a complex interaction between anaesthesia, surgery and extracorporeal circulation (ECG). It has also been shown that each compound used in DAO can individually reduce the inflammatory response to ECG and respiratory complications, and improve postoperative cognitive function and operative haemodynamic stability (3-5). In addition, the absence of opioid use avoids the side effects associated with their use (3-5).<br>Thus, the hypothesis of the present study is that DAO during cardiac surgery is associated with:<br>- Improved intraoperative haemodynamic stability<br>- A reduction in the incidence of postoperative complications<br>- Reduced length of stay in intensive care and in **hospital**
**Research objectives**	**MAIN OBJECTIVE**:<br>To compare the effect of balanced general anaesthesia without opioid versus standard anaesthesia (with opioid) on the occurrence of at least one serious postoperative complication at D30 following cardiac surgery with CPB.<br><br>Postoperative complications are defined in accordance with international recommendations (SFAR, ESICM).<br><br>**SECONDARY OBJECTIVES**:<br>Compare the 2 strategies in terms of :<br>- Occurrence of each type of serious complication in the first 30 days following surgery<br>- Opioid consumption in the 48 hours following extubation

	- Quality of postoperative analgesia during the first 48 hours postoperatively<br>- Quality of post-surgical recovery on discharge from the intensive care unit<br>- Occurrence of arrhythmia by atrial fibrillation and/or atrial flutter<br>- Occurrence of vasoplegic syndrome<br>- Occurrence of postoperative infectious episodes<br>- Time to extubation in intensive care (hours)<br>- Occurrence of non-serious side effects associated with opioid: nausea, vomiting, etc.<br>- Post-operative length of stay in intensive care unit<br>- Total post-operative hospital stay (in days) - Mortality at D90<br><br>- In patients included in the CHU Dijon Bourgogne, to compare the rate of markers of inflammation and bacterial translocation between the 2 types of anaesthesia during samples taken in routine care.
**Study evaluation criteria**	**MAIN EVALUATION CRITERIA**<br><br>The primary endpoint is a composite endpoint corresponding to the occurrence, within 30 days post-operatively, of at least one of the following post-operative complications, defined according to European standards (6):<br><br>- Postoperative neurological dysfunction: ○ Resuscitation delirium assessed using the Confusion Assessment Method for the Intensive Care Unit (CAM-ICU) (7), Search for stroke on CT scan documentation<br>- And/or acute renal failure defined by an increase of at least 50% and/or 26.5 micromol/l in postoperative creatinine compared with the preoperative basal value and/or a diuresis of less than 0.5 ml/kg/h on 6 hours (KDIGO International Society of Nephrology definition) (71)<br>- And/or acute respiratory failure ○ Leading to non-invasive ventilation or optiflow, oro-tracheal intubation for more than 24 hours, or oro-tracheal re-intubation, (6) Acute Respiratory Distress Syndrome, ARDS (Berlin definition: defined by polypnoea > 25, involvement of accessory respiratory muscles or pH < 7.25 and "ALI" defined by respiratory distress with a PaO / FiO_{22} < 300 mmHg/PaO_2 < 80 mmHg ratio under high concentration mask or adult respiratory distress syndrome defined by respiratory distress with a PaO /FiO_{22} < 200 mmHg ratio) (71)<br>- And/or cardiovascular complications: Cardiogenic shock defined by treatment with an inotropic agent (dobutamine, milrinone, levosimendan, adrenaline) (6,8), Acute vasoplegic circulatory failure defined by treatment with vasopressors (adrenaline, noradrenaline, neosynephrine, vasopressin), Postoperative myocardial damage: elevation of troponin I more than 10 times the 99th percentile (4th universal definition of myocardial infarction), Myocardial infarction defined by an increase in cardiac enzymes (troponin I more than 10 times the 99th percentile) plus the appearance of a new Q wave, and/or ST segment elevation and/or a repolarisation disorder on the ECG, and/or an abnormality in the kinetics on echocardiography, and/or an abnormality on coronary angiography (4th universal definition of myocardial infarction), Arrhythmia requiring treatment (ventricular tachycardia, ventricular fibrillation)<br>- And/or death at D30.<br><br>**SECONDARY EVALUATION CRITERIA**<br>- Occurrence of each type of serious complication (see 3.3) within the first 30 days following surgery.

	• Total quantity of opiates (in milligrams) in the 48 hours (D2) following surgery<br>• Number of episodes of postoperative pain (VAS>3) at rest and on coughing in the 48 hours after extubation.<br>• Post-surgical recovery by questionnaire (QoR15) (9,10) on discharge from the intensive care unit<br>• Number of patients treated with a postoperative vasopressor (noradrenaline, vasopressin) within 30 days of surgery<br>• Number of patients with an atrial rhythm disorder (arrhythmia by atrial fibrillation or atrial flutter) within 30 days of the operation<br>• Number of patients presenting with an infectious episode (pneumonia, urinary tract infection, surgical site infection, bacteraemia) in the 30 days following surgery<br>• Time to extubation of the patient in intensive care (hours)<br>• Number of episodes of post-operative nausea and vomiting in the 48 hours following extubation<br>• Number of days spent in intensive care between surgery and first discharge from postoperative intensive care<br>• Number of hospital days between surgery and first discharge from hospital<br>the hospital<br>• Vital status at 90 days post-op (65)<br><br>For patients included in Dijon :<br>➢ Additional samples taken during routine care :<br>- a lipid profile<br>- determination of LPS by mass spectrometry<br>- LPS activity in LAL<br>- fluorometric assay of phospholipid transfer protein (PLTP) activity<br>- measurement of cytokines, CRP and PCT<br>- measurement of I-FABP, GLP-1, Citrulline, Zonuline. ➢ At different times:<br>Before surgery ○ At the end of the bypass ○ 4 hours after bypass ○<br>Day 1 after surgery ○ Day 2 after surgery<br><br>An EDTA tube (4 ml) is taken at different times. It is centrifuged to separate the pellet from the plasma. The plasma is recovered and aliquoted and stored at -80°C in the UMR1231 laboratory freezer. Samples are identified by an anonymous code based on the randomisation number and the sampling time.
**Number of participants expected**	320 participants are expected to take part in this research<br>Breakdown by group: 160 OFA group and 160 control group

**Selection of subjects**	• **INCLUSION <u>CRITERIA</u>**<br>- Patient has given written, free and informed consent<br>- Patients over 18<br>- Patients scheduled for cardiac surgery<br>a. Scheduled<br>b. With CPB<br>c. Types: aortic valve surgery, mitral valve surgery, tricuspid valve surgery, atrial myxoma, coronary artery bypass grafting, aortic surgery, combined surgery<br><br>• **NON-INCLUSION <u>CRITERIA</u>**<br>- Patient not affiliated to or not benefiting from a social security scheme<br>- Patient under legal protection (curatorship, guardianship)<br>- Person subject to a legal protection measure<br>- Pregnant, parturient or breast-feeding women<br>- An adult who is incapable or unable to give consent
	- Patient included once in the study<br>- Patients requiring emergency surgery within 24 hours<br>- Patients who are hypersensitive to local anaesthetics or opiates or to any of the excipients present in the products used.<br>- Patients taking antidepressants, neuroleptics such as non-selective MAOIs (iproniazid), selective A MAOIs (moclobemide), selective B MAOIs (selegiline), gabapentin (Neurontin®), etc.<br>- Patients with untreated atrioventricular conduction disorder<br>- Patient with prolonged QTc (> 450 ms) on preoperative ECG<br>- Patients with severe hepatic impairment (TP<30%)<br>- patients suffering from respiratory insufficiency (patients on long-term oxygen therapy, except those with OSA)<br>- Patients with uncontrolled epilepsy<br>- Patient with preoperative cognitive dysfunction (MMS <24)<br>- Patient with intracranial hypertension<br>- Patients with chronic renal failure (dialysis, creatinine > 200 µmol L) $^{-1}$<br>- Patients with porphyria<br>- Patient treated with linezolid (Zyvoxid®)<br>- Patients with severe arterial hypotension (SAP<90 mmHg)
**Assessment of expected benefits and risks**	Better overall intraoperative management of the patient, as well as better postoperative management of analgesia and a reduction in the incidence of postoperative complications,<br>- Reduction in opioid-related side effects such as nausea and vomiting or episodes of bradypnoea,<br>- Improvement in post-operative cognitive function,<br>- Reduced length of stay in intensive care and hospital,<br>This protocol is part of a global approach aimed at improving perioperative care, such as assisted recovery programmes after surgery. Cardiac surgery remains a high-risk surgery for which we need to improve our practices, because the incidence of complications, the length of hospital stay and the costs associated with these treatments are high.<br>Reducing the incidence of perioperative complications and the length of stay in intensive care and in hospital would also represent a saving in terms of human and material resources, and therefore an advantage in terms of public health.

**Conduct of the study**	- CHRONOLOGICAL DESCRIPTION OF THE STUDY<br>1. Information for research participants<br>2. Obtaining free, informed and written consent<br>3. Inclusion<br>4. Randomisation<br>5. Anaesthesia performed according to group (control, OFA)<br>6. Evaluation of primary endpoint<br>7. Evaluation of secondary endpoints<br>8. Output of the study<br><br>One of the investigators will provide information about the study protocol during the anaesthetic consultation (between 48 hours and 2 months before cardiac surgery). Written informed consent will be collected during the pre-operative visit (baseline visit) the day before or the morning of the operation. Inclusion and non-inclusion criteria will be checked before final inclusion. At the baseline visit, demographic and surgical data will be collected.<br>Randomisation will take place on the day of cardiac surgery. Participants will be randomised in a 1:1 ratio to receive either opioid-free anaesthesia (interventional group: OFA) or conventional anaesthesia, including opioid (control group). This allocation will be stratified according to centre and Euroscore 2 (<3, ≥3). During cardiac surgery, respiratory and haemodynamic management will be identical in the 2 groups, and will correspond to the department's usual practices. Only the management of drugs during anaesthesia differed between the 2 groups.<br>Post-operative pain at rest and with coughing (VAS) and the onset of nausea/vomiting were recorded at 48 hours after extubation. At D2, after surgery,

	total opioid consumption will be recorded. The occurrence of serious adverse events will be recorded up to D30.<br>Vital status at D30, complications between D7 and D30 and the length of stay in intensive care and hospital will be collected on the day of the follow-up visit carried out between D30 and D60 (+/- 7 days) postoperatively (in hospital or by telephone) and by telephone at D90 (+/- 7 days) in order to obtain the length of stay in intensive care and hospital.<br>Due to the nature of the operation, it is not possible to carry out a blind operation, but post-operative management will be carried out blind to the allocated treatment (different person not informed).
**Collection of biological samples**	☐ No collection<br><br>☐ Creation of a collection of biological samples Use of these biological samples<br>☐ within the framework of the project with destruction at the<br>use of these biological samples for the purposes of this research,<br>☒ conservation and future use of this collection<br><br>Shelf life beyond study: while stocks last<br>Place of storage:☐ CRB du CHU Dijon Bourgogne;☒ other: INSERM UMR 1231 laboratory
**Treatments and products used**	Experimental treatment :<br>- Lidocaine hydrochloride anhydrous Associated treatments :<br>- Sufentanil<br>- Dexamethasone<br>- Ketamine<br>- Propofol<br>- Cisatracurium or **atracurium**

**Dosage, route of administration and duration of administration**	Dosage :<br>- Lidocaine 1.5 mg Kg^{-1} bolus then 1.5 mg Kg^{-1} h^{-1} until sternotomy then 1 mg Kg^{-1} h^{-1} until end of operation<br>- Ketamine 0.5 mg Kg^{-1} for anaesthetic induction<br>- Dexamethasone 0.1 mg Kg^{-1} at anaesthetic induction<br>- Magnesium sulphate 30 mg Kg^{-1} for anaesthetic induction<br>- Sufentanil in "TIVA" for a blood target of between 0.35 and 0.5 ng ml^{-1} , stopped at the end of the procedure<br>- Propofol in « TIVA" for a BIS target between 40 and 60<br>- Intravenous cisatracurium (0.15 mg Kg^{-1}) or atracurium with curameter monitoring.<br>Route of administration: intravenous<br>Duration of administration: the time required for general anaesthesia, i.e. 2 to 7 hours.
**Statistical analysis**	**Sample size <u>calculation</u>**<br>Assuming a frequency of complications in the standard group of 60% (BMC 2019 GUINOT) and a reduction of 20% in the OFA group (minimal difference expected given the simultaneous consideration of several clinical events), 144 patients per group are required, i.e. a total of 288 patients with a power of 90% and an alpha risk (two-sided) of 5%. Given the composite nature of the primary endpoint, we opted for 90% power. Anticipating 10% non-evaluable data for the primary endpoint, 320 patients will be included.<br>**<u>Analysis of primary endpoint</u>:**<br>The rate of post-operative complications in the two treatment groups will be compared using logistic regression adjusted for the stratification factors (centre and Euroscore 2).

**Expected results and alternatives**	1. Improved intraoperative stability with less use of vasopressor agents<br>2. Improved post-operative outcomes for patients, with a reduction in post-operative cognitive disorders and renal failure,<br>3. Reduction in postoperative opioid consumption<br>4. Reduced incidence of complications<br>5. Reduced length of stay in intensive care and in hospital
**Provisional timetable**	- length of inclusion period: 30 months<br>- total duration of participation for a person taking part in the research: 90 days<br>- total duration of the study (inclusion + possible follow-up): 33 months<br>Period of exclusion for the person: no<br>Period of exclusivity for the person: yes<br>The patient cannot take part in other research evaluating surgical techniques

Protocol: OFACAR Version: 3 Date: 04/01/2022

DIAGRAM OF THE STUDY

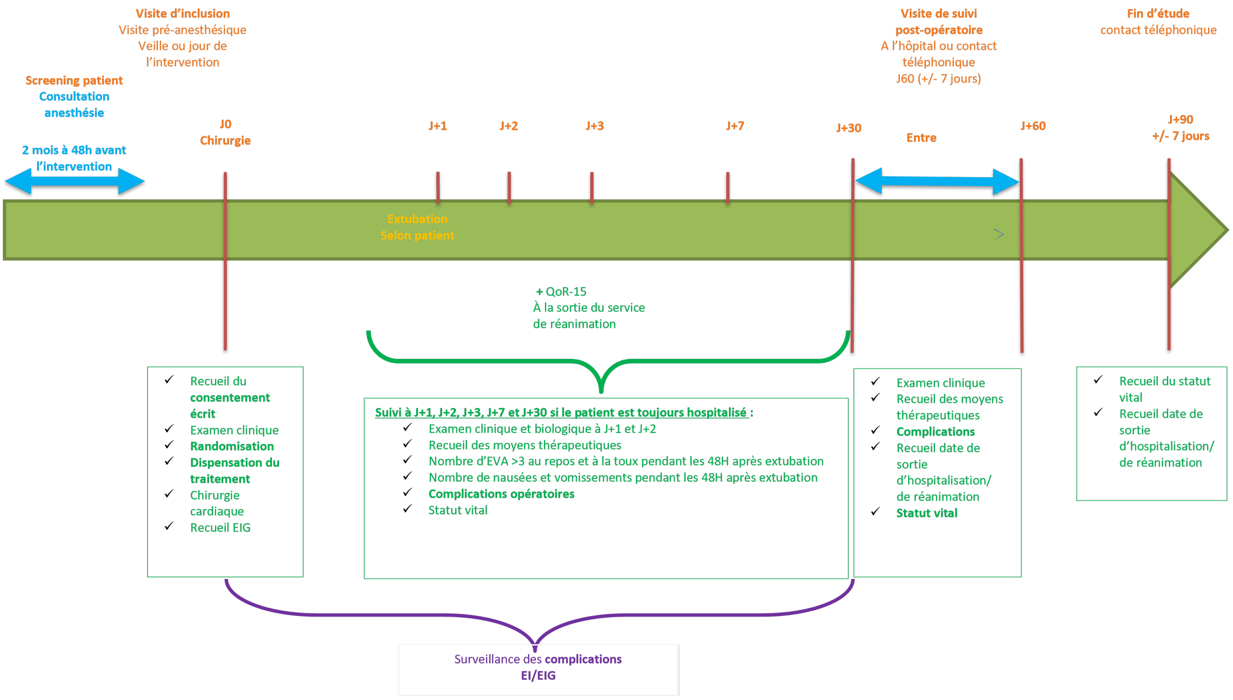

TABLE OF CONTENTS

INVESTIGATORS AND ADMINISTRATIVE STRUCTURE OF THE STUDY 2
SYNOPSIS 4
LIST OF ABBREVIATIONS AND DEFINITION OF TERMS 14
1. STATE OF THE ART AND RATIONALE FOR THE STUDY 15
1.1 Current scientific knowledge and outstanding questions 15
1.1.1 Anaesthesia - From the introduction of opioids... 15
1.1.2 Opioid-free anaesthesia 16
1.1.3 Data on anaesthesiology and complications in cardiac surgery..... 19
1.2 Research hypothesis 20
1.3 Originality of the project and expected results 20
2. OBJECTIVES 20
2.1. Main objective 21
2.2 Secondary objectives 21
3 ASSESSMENT CRITERIA... 21
3.1 Primary endpoint.. 21
3.2 Secondary assessment criteria 22
3.3. Definition of complications according to the European Society of Anaesthesia and Intensive Care 22
4 METHODOLOGY 24
4.1. Diagram of the study 24
4.2 Calculating the number of subjects required... 24
4.3. Study population 24
4.3.1 Inclusion criteria.. 24
4.3.2 Non-inclusion criteria 24
4.3.3 Premature and definitive study exit criteria................................... 25
4.3.4 End of protocol study.. 25
4.4 Project feasibility 25
4.5. Benefit-risk balance 25
5. TREATMENTS.. 26
5.1. Experimental treatment.. 26

5.2. Treatment circuits 26
5.2.1. Labelling of treatments 26
5.2.2. Packaging and blinding of treatments (if applicable) 26
5.2.3. Storage 27
5.2.4. Dispensing treatments 27
5.2.5. Accounting and compliance 27
5.2.6. Return and destruction of products 27
5.3. Associated treatments 27
5.4. Unauthorised treatments 28
5.5. Management protocol 28
6. progress of the study 30
6.1. Pre-selection of patients 30
6.2 Information and obtaining consent 31
6.4. Randomisation procedures 31
6.5. Monitoring procedures / questionnaires 31
6.6. For patients included at the CHU Dijon Bourgogne : 32
6.7. End of the study 32
6.8. Technical description of measured parameters 32
6.9. Description of permanent or temporary shutdown rules 33
6.10. Period of exclusion - exclusivity 33
6.11. Duration of the study 33
6.12. Study organisation chart 34
7. DATA MANAGEMENT 36
7.1. Confidentiality 36
7.2. Collection of data 36
7.3. Quality and Safety Manager 38
7.4. Storage and archiving 38
8. COORDINATION OF THE STUDY 38
8.1. Steering committee 38
8.2. Independent Supervisory Committee 39
9. SAFETY ASSESSMENT 39

9.1. Description of safety assessment parameters 39
9.2 Adverse events and reactions, definitions.. 39
9.2.1. Definitions 39
9.2.2. Research events.. 40
9.2.3. Responsibilities of the investigator 40
9.2.4. Role of the promoter.. 41
10 STATISTICAL ANALYSES 42
10.1 Analysis population 42
10.2 Choice of statistical tests 42
10.3 Analysis of missing data:... 43
10.4 Choice of analysis software 43
10.5 Significance threshold... 43
10.6 Person responsible for the analysis and place of analysis 43
11 ethical and regulatory aspects 43
11.1 Ethical conduct of the study 43
11.2 Responsibilities of the investigator... 44
11.3 Responsibilities of the promoter.. 44
11.3.1 Personal Data Protection Committee and Competent Authority.... 44
11.3.2 Protection of personal data.. 45
11.3.2.1 CNIL 45
11.3.2.2 Confidentiality 45
11.3.3 Insurance/Compensation for damage to patients 45
11.3.4 Authorisation, persons responsible and study sites...................... 46
11.3.5 Informing the management and pharmacies of participating centres.. 46
11.3.6 Informing subjects of overall research results 46
11.3.7 Audit and inspection.. 46
11.3.8 Archiving 46
12 FINANCING THE STUDY... 46
14. REPORTS AND PUBLICATIONS 46
13.1. Final report.. 46
13.2. Publication rules.. 47

14. BIBLIOGRAPHY 47

LIST OF ABBREVIATIONS AND DEFINITION OF TERMS

**Abbreviations**	**Meanings**
AC	Competent Authority
CEC	Extracorporeal Circulation
PPC	Individual Protection Committee
CRF	Case Report Form
CTCAE	Common Terminology Criteria for Adverse Event
ECG	ElectroCardioGramme
ECMO	Extracorporeal Membrane Oxygenation
EIG	Serious Adverse Event
EVA	Visual Analogue Scale
ARDS	Acute respiratory distress syndrome

1. STATE OF THE ART AND RATIONALE FOR THE STUDY

1.1 Current scientific knowledge and outstanding questions

1.1.1 Anaesthesia - From the introduction of opioids...

Since the birth of anaesthesia, its aim has remained the same, providing hypnosis, analgesia and muscle relaxation for the performance of surgery and the well-being of the patient. Prior to the introduction of opioids in the 1960s, anaesthesia was achieved by deep inhalation of a high dose of hypnotics to achieve hypnosis and immobility, and to suppress the sympathetic response to pain (1). These high doses often led to haemodynamic instability.

The introduction of synthetic opioids made it possible to reduce the dosage of hypnotics, significantly improving haemodynamic stability. Thus, since the late 1960s, the systematic administration of opioid derivatives has been considered one of the pillars of modern general anaesthesia. The opioid derivatives used during anaesthesia are synthetic derivatives characterised by their extreme potency and short duration of action compared with the reference opioid analgesic (1). The use of these opioid derivatives has become widespread with the development of administration systems based on a "target concentration", and the appearance of monitoring systems assessing the nociception-antinociception balance. All this, without the very principle underlying the administration of opioid derivatives during anaesthesia ever being called into question.

The management of general anaesthesia has evolved over time but is still based on the use of a hypnotic (intravenous or inhaled agent) combined with the use of a opioid derivative (usually sufentanil) (11). Since the 1990s, the term "balanced general anaesthesia" has been used. The opioid derivative is most often used in high doses because of its potential cardioprotective effects (protection against myocardial ischaemia, etc) (12-14). However, balanced general anaesthesia is associated with numerous haemodynamic events (arterial hypotension, use of vasopressor agents) (15,16). With the development of new techniques for monitoring depth of anaesthesia and nociception, these old concepts about the concomitant use of high doses of hypnotic and opioid agents are gradually being reexamined (17). For example, Forestier et al have demonstrated that the use of an algorithm incorporating BIS measurement enables hypnotic and opioid agents to be titrated (17). Despite this progress, the use of balanced general anaesthesia is still associated with a number of events (haemodynamic instability, confusional disorders, acute renal failure, etc.) (15). These events are thought to be related to the specific effects of the anaesthetic and/or opioid agents (isolated or synergistic effect) and/or to their secondary effects (cardiovascular, respiratory, etc.).

Although opioid analgesics are used on a daily basis, there are a number of significant side effects. An illustration of the problems associated with the use of opioid analgesics is the health crisis in the United States of America, linked to increased consumption of opioid analgesics by a significant proportion of the population. This over-consumption is thought to be linked to inappropriate peri-operative prescriptions of opioid analgesics (4), and is independent of the type of surgery (3). The North American government has just declared a health emergency by redistributing funds to manage this crisis. It is therefore a public health problem. Overall, it seems necessary to review clinical practices relating to the prescription and use of opioid analgesics in the perioperative period.

Problems associated with the use of opioid during surgery

There are several aspects to this problem.The use of synthetic opioid derivatives during anaesthesia is associated :

1. to lasting hyperalgesia postoperatively (5). This observation is supported by numerous experimental studies demonstrating that opiates cause both analgesia and hyperalgesia, even with a single dose, and all the more so in the presence of an inflammatory phenomenon (5,18,19). This hyperalgesia lasts much longer than analgesia (19),
2. addiction, leading to overconsumption of analgesics, including opioid (20),

3. to a number of phenomena: respiratory depression, increased sleep apnoea syndrome, pruritus, constipation, nausea and vomiting (1,21,22). These phenomena have been shown to be associated with perioperative morbidity and mortality (23).
4. the occurrence of postoperative confusional disorders, which increase the length of hospital stay (24). The older the patient and the more major the surgery, the greater the risk of confusion (24,25),
5. a number of immune phenomena with possible immunosuppression. Several retrospective studies appear to show an association between the use of these opiates and recurrence of cancer (26,27). Endogenous opiates are effectors of the immune response at multiple levels. Opiates can interfere with the immune system: primary and anti-tumour immunity. Questions persist as to their possible involvement in cancer recurrence, particularly with the use of high doses of opioid derivatives (26,27).
6. All these effects result in additional financial costs for patient care (28).

In summary, the use of opioid derivatives is associated with a certain number of side effects which may limit the improvement in general anaesthetic practices and the reduction in perioperative complications.

1.1.2 Opioid-free anaesthesia

For several years, a number of doctors have been developing a concept of anaesthesia without opioid analgesia. Prof Huguenard and Prof Laborit had put forward the hypothesis that "states of shock result from a kind of neurovegetative delirium detached from its cause, linked to sympathetic-catecholaminergic hyperreactivity, defensive in the first instance and in various circumstances, by supporting central haemodynamics, but then liable to damage deep tissue life by excess, exhaustion and inversion" (29). This concept gave rise to the principle of using a drug synergy to modulate all the physiological responses (neuro-vegetative system, anti-nociception, haemodynamics, etc.) to surgery (29). With the development of new opioid analgesics and sedatives with more manageable pharmacokinetics, this concept has fallen into disuse in favour of balanced general anaesthesia using a opioid and a hypnotic.

Research and the development of new therapeutic classes of analgesics (alpha blockers, ketamine, local anaesthetics....) and increased knowledge of perioperative pathophysiology (neurovegetative, nociceptive) have highlighted a whole range of problems associated with the use of opioid derivatives (arguments developed previously) and opened up new possibilities for managing this perioperative pathophysiological response without recourse to the use of a opioid analgesic. In this sense, since the 90s the concept of opioid sparing and then of balanced general anaesthesia without opioid has been developed (OFA) (29,30).

The principle of OFA is based on the fact that, in a sleeping patient, a sympathetic reaction marked by haemodynamic changes does not reflect a painful phenomenon, that a painful phenomenon in a sleeping patient is not memorised, and that hormonal stress, the sympathetic reaction and the inflammatory reaction can be controlled by therapeutic classes other than a opioid analgesic agent (30). OFA is based on the use of several molecules with the aim of having an analgesic effect (lidocaine, dexamethasone, ketamine, magnesium), reducing the sympathetic response (beta-blocker, lidocaine), reducing hormonal stress (beta-blocker, lidocaine, corticoid) and the inflammatory response (lidocaine, dexamethasone, ketamine, beta-blocker). Although this approach to anaesthesia is regularly used in clinical practice, it remains a novel approach. The literature on this type of anaesthesia consists mainly of observational studies or rare prospective studies involving small groups of patients, the main aim of which is usually to demonstrate the feasibility of the technique in the surgeries evaluated (31-33).

The main results of this work show :

1. The perioperative safety profile of this approach. OFA is not associated with more deleterious haemodynamic events. On the contrary, the trend is towards better haemodynamic stability with less use of vasopressor agents and maintenance of blood pressure (31,32,34,35).

2. The user-friendliness of the published protocols. They are easy to implement and safe.
3. The positive effect on the respiratory system, particularly in bariatric and cardiac surgery, with a reduction in postoperative apnoea episodes, respiratory insufficiency, recourse to non-invasive ventilation, and an improvement in oxygen saturation (31,32).
4. The positive effect on intraoperative and postoperative analgesia. OFA is significantly associated with a reduction in opioid consumption and opioid-related side-effects (pain, hyperalgesia) (31,32,34,36-41).
5. The positive effect on post-operative vigilance. Patients are less confused and more present
(31).

Pharmacological and safety data on compounds used in DAO

We will only describe the molecules used in this protocol. The description below summarises the studies which have investigated the individual effect of each compound in standard general anaesthesia (excluding OFA).

Dexamethasone

Dexamethasone is a potent corticosteroid with no mineralocorticoid effects. Dexamethasone is recognised as an effective antiemetic agent when administered at a dose of 50 µg kg^{-1} during induction. It has analgesic activity at higher doses (100 µg kg^{-1}). Several studies have demonstrated a reduction in pain scores and the incidence of nausea and vomiting. This effect is thought to be related to its anti-inflammatory action at the surgical site, with a reduction in oedema and local mechanisms of hyperalgesia (42). Dexamethasone is administered prior to incision to enhance its analgesic effect. The optimal dose is suggested to be 8 to 10 mg (42).

Beta-blocker

Esmolol is an ultra-short-acting, cardio-selective beta1-adrenergic receptor antagonist that is rapidly hydrolysed by red blood cell esterases. It has a short clinical duration of action of around 9 minutes (43,44). The benefits of beta-blockers in cardiovascular disease are well established, as they can reduce the sympathetic response (arterial hypertension, sinus tachycardia). It also has an analgesic effect, which some authors have suggested as an alternative to intraoperative opioids (44-46). Studies have shown that the use of esmolol is associated with a reduction in opioid analgesic consumption, the incidence of postoperative nausea and vomiting, and improved postoperative rehabilitation (44-46). This work has been carried out in several types of abdominal surgery and also in pregnant women, with a good safety profile. This effect is thought to be linked to an analgesic effect of esmolol. Esmolol also reduces the concentration of intravenous or inhaled hypnotic agents during anaesthesia (47). Esmolol can be used as a continuous infusion or as an iterative bolus. Several protocols have been described. The titration dose is 1 µg to 0.5 mg Kg^{-1} , with maintenance at an average dose of 5 to 250 µg Kg min^{-1} .

Lidocaine

Lidocaine is a local anaesthetic with anti-arrhythmic activity (class 1B) by blocking sodium channels (48-54). This molecule provides good local or perineural anaesthesia and also analgesia when administered intravenously during surgery (47,49). Lidocaine has been shown to have sedative, analgesic, antihyperalgesic and anti-inflammatory activities (attenuation of leukocyte-platelet activation, etc.). More specifically in cardiac surgery, lidocaine is thought to have a neuroprotective and cardio-protective effect during ischaemia-reperfusion (54,55).

Numerous studies in abdominal surgery have confirmed the anti-hyperalgesic, analgesic and sedative properties and safety profile of lidocaine(48-54). Lidocaine administered intravenously during surgery also reduces intraoperative hypnotic and opioid consumption and improves haemodynamic stability (53). These effects are associated with a postoperative reduction in pain (rest and effort), opioid consumption, the incidence of nausea and vomiting, and the duration of ileus, and therefore a reduction in the length of hospital stay.

(53). A Cochrane review found a reduction in postoperative pain over the first 24 hours, the more marked the invasive the surgery, with a beneficial effect on gastrointestinal recovery, analgesic requirements, nausea and length of hospital stay, PONV and opioid requirements (56). It also confirms its good safety profile, with a very low incidence of side effects (56).
In cardiac surgery, lidocaine has been studied for its anti-inflammatory, neuroprotective and cardioprotective effects (54,55,57-59). This suggests that lidocaine administered during surgery could reduce the number of patients with postoperative cognitive dysfunction by reducing cerebral inflammation secondary to surgery and cardiopulmonary bypass (CPB) (57-59).

Safety and plasma levels
It has been shown that therapeutic levels are between 2 and 5 µg ml^{-1} , and that signs of toxicity appear at higher plasma levels: 6 to 10 µg ml^{-1} (60). In general, the protocol is 1-1.5 mg kg^{-1} in bolus at anaesthetic induction with a maintenance dose of 2-3 mg min^{-1} or 1-1.5 mg Kg^{-1} h^{-1} up to 24 h postoperatively. This protocol is widely used and has been shown to be safe. Several studies, including some carried out in cardiac surgery with CPB, confirm the safety profile of continuous intraoperative administration of lidocaine at a maximum dose of 3 mg Kg^{-1} h^{-1} (Table). These studies found mean plasma concentrations of 2 µg ml^{-1} , well below the toxicity threshold of 5 µg ml^{-1} .

Table: Serum concentrations according to intraoperative dosage regimens.

	Safety < 5 µg ml^{-1}	Duration	Plasma concentration (µg ml) $^{-1}$
Groudine et al, 1998 (50)	Bolus: 1.5 mg kg^{-1} Maintenance: 3 mg min^{-1}		1,3-3,7
Koppert et al, 2004 (38)	Bolus: 1.5 mg kg^{-1} Maintenance: 1.5 mg kg^{-1} h^{-1}	6 h	1,9 +/- 0,7
Kaba et al, 2007 (51)	Bolus: 1.5 mg kg^{-1} Maintenance: 2 mg kg^{-1} h^{-1}	24 h	1.6 +/- 0.9 at 5 min<br>1.8 +/- 0.5 to 1h<br>2.7 +/- 1.7 to 24h
Herroeder et al, 2007 (40)	Bolus: 1.5 mg kg $^{-1}$ Maintenance: 3 mg min $^{-1}$	24 h	1,1-4,2
Landow et al (52)	Bolus: 1.5 mg kg $^{-1}$ Maintenance: 2 mg min^{-1}	6h during CPB	1,7-2,7
Yung-Wei Hsu et al 2011(53)	Bolus 1 mg kg^{-1} then 4 mg min^{-1} ,2 mg min^{-1} , 1 mg min $^{-1}$	46 h	2.32 ± 0.63 end of surgery<br>2,56 ± 0,86 24 h<br>3.34 ± 0.91 µg ml^{-1} 48h

Ketamine
Ketamine is an intravenous anaesthetic with anti-hyperalgesic and analgesic properties (61-63). The effects of ketamine depend on its plasma concentrations. The analgesic effect persists as long as plasma concentrations remain above 100-200 ng ml^{-1} , concentrations ten times lower than those required for narcosis. Anti-hyperalgesic effects occur at lower concentrations, probably as low as 20 ng ml^{-1} , at which concentrations ketamine no longer has any analgesic effect, but potentiates the analgesia caused by opioid analgesics. In rats, ketamine, at non-analgesic doses, reduces the acute tolerance and hyperalgesia observed after administration of alfentanil, and then proposed a classification of doses administered:

- analgesic effect on acute, postoperative pain, at "subanaesthetic" doses (0.5 mg kg), $^{-1}$
- antiallodynic" effect on subacute or chronic pain, at "subanalgesic" doses (0.25 mg.kg), $^{-1}$
- antihyperalgesic effect (and attenuation of opioid tolerance), for the lowest doses (0.07-0.15 mg kg^{-1}), at which ketamine has no direct analgesic effect.

Numerous studies have shown that the use of low-dose ketamine as an adjuvant to opioid analgesics reduces pain intensity at 6, 12, 24 and 24 hours.
48 hours post-operatively (62). The addition of ketamine to PCA opioid in a 1:1 ratio with an 8 min lock-in interval has also been described with positive effects after major orthopaedic procedures. All studies conclude that the perioperative use of ketamine is safe (62). In cardiac surgery, ketamine is thought to have neuroprotective effects by reducing the inflammatory response. One study found a reduction in the incidence of post-operative cognitive dysfunction with a single dose of 0.5 mg/kg^{-1} at induction (63).

1.1.3 Data on anaesthesiology and complications in cardiac surgery

Cardiac surgery is a type of surgery where, by definition, the patients are carriers of cardiac pathologies (coronary, valvulopathy, ventricular dysfunction or congenital disease). They are usually elderly and have numerous co-morbidities (15,64), and are therefore classified as ASA III or IV. Their frailty is such that most of them would be rejected for general surgery. They therefore require extensive management with close, multiparametric monitoring (64). Although their basic functional capacity still seems satisfactory, these fragile patients have lost all reserve capacity, because their maximum functional capacity has collapsed (65). They can no longer compensate for the slightest alteration in their haemodynamic, respiratory or neurological conditions. Major surgery can be performed on these patients with a high success rate, provided that homeostasis is rigorously maintained. Anaesthesia, surgery and extracorporeal circulation (ECG) must be performed without the slightest incident. The smallest incident can have a major impact, with complications. This is why this surgery is considered to be major surgery, with a high incidence of postoperative complications, which fall into several classes (cardiovascular, respiratory, renal, digestive, neurological, infectious, haematological, etc.) (15,66). The cumulative incidence of these complications can be as high as 60% of patients operated on (15,66,67). As a result, research in cardiac surgery into strategies aimed at reducing the occurrence of these complications is important. Furthermore, the association of this extremely fragile patient population with certain specific surgical techniques, such as CPB, means that cardiac surgery is a separate entity in the field of clinical research in anaesthesia, for which the results of studies carried out in general surgery cannot be generalised, and which therefore requires its own evaluation.

The literature offers some data on the beneficial effects in non-cardiac surgery and on the expected benefits of each molecule used in the context of OFA in cardiac surgery (see above). To date, no randomised study (search of databases of indexed studies, PHRC, and databases such as clinicaltrial.gov) has evaluated the effect of OFA in cardiac surgery on a postoperative criterion including postoperative complications and/or patient well-being (analgesia, cognition). A retrospective non-randomised study by our group demonstrated the feasibility and reduced incidence of a composite endpoint and analgesic requirements with the use of OFA in cardiac surgery (68). To date, only one randomised study has been carried out in non-cardiac surgery with the aim of evaluating the impact of OFA on immediate postoperative respiratory complications and analgesia (2). The study was stopped early because of side effects associated with the use of dexmetomidine (conductive and rhythmic disorders). The results are awaiting publication. We are specifically interested in cardiac surgery because it is a major operation, most often involving an elderly population, with a high incidence of postoperative complications, for which we need to improve our practices. The occurrence of these complications is the result of a complex interaction between the different aspects (anaesthesia, surgery, CPB) of the perioperative management of patients. As developed previously, the molecules used in OFA and the absence of a opioid derivative suggest that this approach could improve the management of cardiac surgery patients at several levels (hypoperfusion, inflammation, respiratory, neurocognitive).

What are the risks of an OFA in cardiac surgery?

The risks are associated with the use of intravenous lidocaine during the anaesthetic period. A large number of studies have validated the use of lidocaine in cardiac surgery with CPB and in non-cardiac surgery for periods of up to 48 hours (48-60). The dosages proposed in this protocol are associated with serum concentrations well below the toxicity thresholds, and the duration of administration is limited to general anaesthesia (see table above). This toxicity is neurological and cardiac in nature. However, it can be reversed by intravenous administration of intralipid, which must be available in every operating theatre (69). Apart from the risks associated with overdose, no other risks are expected. One risk could be of a cardiac/rhythmic nature with the use of dexmetomidine, which we do not use in this protocol.

1.2 Research hypothesis

We hypothesise that the reduction in opioid consumption combined with the specific effects (analgesic, anti-inflammatory, haemodynamic stability, etc.) of the molecules used in OFA would make it possible to reduce post-operative complications in cardiac surgery.

We can therefore hope to demonstrate in the OFA group :

- A reduction in postoperative pain assessed by the VAS at rest and with coughing, and in the total dose of opioid consumed.
- A reduction in the incidence of post-operative cognitive disorders: confusional syndrome, vigilance disorders, delirium, etc.
- A reduction in the intraoperative inflammatory response.
- A reduction in complications linked to ischaemia-reperfusion, inflammation and organ hypoperfusion: myocardial ischaemia, ventricular rhythm disorders, use of vasopressor and inotropic agents, renal failure.
- A reduction in the incidence of post-operative complications, particularly those associated with the use of opioid analgesics: length of tracheal intubation, haemodynamic instability, respiratory distress, nausea and vomiting.
- A reduction in the length of stay in intensive care and hospital.

The aim of our study is to demonstrate that balanced general anaesthesia without opioid (BGA) reduces the number of patients with at least one complication.

1.3 Originality of the project and expected results

This is the first randomised study in cardiac surgery to evaluate anaesthesia without opioid analgesics (OFA) compared with usual practice based on balanced general anaesthesia (opioid analgesics and an intravenous hypnotic agent). This study follows on from a retrospective study that demonstrated these effects, with all the drawbacks of a non-randomised retrospective study. This approach is important because it forms part of an overall approach to intraoperative optimisation of cardiac surgery patients in order to reduce the incidence of complications (described previously and in the objectives), and the length of hospital stay. The use of opioid is associated with a number of side effects which in cardiac surgery can increase morbidity and mortality (5,18-28). Among these, two aspects are important: disorders related to intraoperative hypoperfusion and postoperative neurocognitive disorders, which may affect up to 50% of patients (15,54).

The expected results are an improvement in the management of perioperative patients undergoing cardiac surgery with CPB thanks to a reduction in the incidence of complications. A reduction in the incidence of complications is associated with a reduction in the length of hospital stay and the cost of patient management.

2. OBJECTIVES

2.1. Main objective

To compare the effect of balanced general anaesthesia without opioid versus standard anaesthesia (with opioid) on the occurrence of at least one serious postoperative complication at D30 following cardiac surgery with extracorporeal circulation.

Postoperative complications are defined in accordance with international recommendations (SFAR, ESICM) (see primary endpoint) (6).

2.2 Secondary objectives

Compare the 2 strategies in terms of :
- Occurrence of each type of serious complication in the first 30 days following surgery
- Opioid consumption in the 48 hours following extubation
- Quality of postoperative analgesia during the first 48 hours postoperatively
- Quality of post-surgical recovery on discharge from the intensive care unit
- Occurrence of arrhythmia by atrial fibrillation and/or atrial flutter
- Occurrence of vasoplegic syndrome
- Occurrence of postoperative infectious episodes
- Time to extubation in intensive care (hours)
- Occurrence of non-serious side effects associated with opioid: nausea, vomiting, etc.
- Post-operative length of stay in intensive care unit
- Total post-operative hospital stay (in days) - Mortality at D90

- In patients included in the CHU Dijon Bourgogne, to compare the rate of markers of inflammation and bacterial translocation between the 2 types of anaesthesia during samples taken in routine care.

3 ASSESSMENT CRITERIA

3.1 Primary endpoint

The primary endpoint is a composite endpoint corresponding to the occurrence, within 30 days post-operatively, of at least one of the following post-operative complications, defined according to European standards (6):

- Postoperative neurological dysfunction :
  - Resuscitation delirium assessed using the Confusion Assessment Method for the Intensive Care Unit (CAM-ICU) (7)
  - Search for stroke on CT scan documentation
- And/or acute renal failure defined by an increase of at least 50% and/or 26.5 micromol/l in postoperative creatinemia compared with the preoperative basal value and/or a diuresis of less than 0.5 ml/kg/h over 6 hours (KDIGO International Society of Nephrology definition) (71).
- And/or acute respiratory failure ○ Leading to non-invasive ventilation or optiflow, oro-tracheal intubation for more than 24 hours, or oro-tracheal re-intubation (6)
  - Acute respiratory distress syndrome, ARDS (Berlin definition: defined by polypnoea > 25, involvement of accessory respiratory muscles or pH < 7.25 and "ALI" defined by respiratory distress with a PaO /FiO22 < 300 mmHg/PaO2 < 80 mmHg ratio under high concentration mask or adult respiratory distress syndrome defined by respiratory distress with a PaO /FiO22 < 200 mmHg ratio) (71)
- And/or cardiovascular complications ○ Cardiogenic shock defined by treatment with an inotropic agent (dobutamine, milrinone, levosimendan, adrenaline) (6,8).
  - Acute vasoplegic circulatory failure defined by treatment with vasopressors (adrenaline, noradrenaline, neosynephrine, vasopressin)
  - Postoperative myocardial damage: elevation of troponin I more than 10 times the 99th percentile (4th universal definition of myocardial infarction)
  - Myocardial infarction defined by an increase in cardiac enzymes (troponin I more than 10 times the 99th percentile) plus the appearance

of a new Q wave, and/or ST segment elevation and/or a repolarisation disorder on the ECG, and/or an abnormality in the kinetics on echocardiography, and/or an abnormality on coronary angiography (4th universal definition of myocardial infarction).
  - ○ Arrhythmia requiring treatment (ventricular tachycardia, ventricular fibrillation) -
- And/or death at D30.

3.2 Secondary assessment criteria

- Occurrence of each type of serious complication (see 3.3) within the first 30 days following surgery.
- Total quantity of opiates (in milligrams) in the 48 hours (D2) following surgery
- Number of episodes of postoperative pain (VAS>3) at rest and on coughing in the 48 hours after extubation.
- Post-surgical recovery by questionnaire (QoR15) (9,10) on discharge from the intensive care unit
- Number of patients treated with a postoperative vasopressor (noradrenaline, vasopressin) within 30 days of surgery
- Number of patients with an atrial rhythm disorder (arrhythmia due to atrial fibrillation or atrial flutter) in the 30 days following surgery
- Number of patients presenting with an infectious episode (pneumonia, urinary tract infection, surgical site infection, bacteraemia) within 30 days of surgery
- Time to extubation of the patient in intensive care (hours)
- Number of episodes of post-operative nausea and vomiting in the 48 hours following extubation
- Number of days spent in intensive care between surgery and first discharge from postoperative intensive care
- Number of days in hospital between surgery and first hospital discharge
- Vital status at 90 days post-op (65)

For patients included in Dijon :

➢ Additional samples taken during routine care :

- a lipid profile
- determination of LPS by mass spectrometry
- LPS activity in LAL
- fluorometric assay of phospholipid transfer protein (PLTP) activity
- measurement of cytokines, CRP and PCT
- measurement of I-FABP, GLP-1, Citrulline, Zonuline. ➢ At different times: Before surgery, At the end of the bypass , 4 hours after bypass, Day 1 after surgery, Day 2 after surgery

An EDTA tube (4 ml) is taken at different times. It is centrifuged to separate the pellet from the plasma. The plasma is recovered and aliquoted and stored at -80°C in the UMR1231 laboratory freezer. Samples are identified by an anonymous code based on the randomisation number and the sampling time.

3.3. Definition of complications according to the European Society of Anaesthesia and Intensive Care :

These follow the recommendations of the European Society of Anaesthesia and Intensive Care and are assessed over the first 30 days post-op (6).

- **Neurological** :
  - ○ Cerebrovascular accident, defined by CT scan documentation, ○ Resuscitation delirium assessed by the CAM-ICU questionnaire (7).

- **Respiratory** :
  - ○ ARDS : Acute respiratory distress syndrome defined by polypnoea > 25, involvement of accessory respiratory muscles or pH < 7.25 and "ALI"

defined by respiratory distress with a PaO /FiO22 ratio < 300 mmHg/PaO_2 < 80 mmHg under high concentration mask or adult respiratory distress syndrome defined by respiratory distress with a PaO /FiO22 < 200 mmHg (71)
- ○ Prolonged oro-tracheal intubation lasting more than 24 hours.
- ○ Non-invasive ventilation or optiflow ○ Tracheal reintubation.

- **Cardiovascular** :
  - ○ Cardiogenic shock requiring inotropic treatment (adrenaline, dobutamine, phosphodiesterase inhibitor, levosimendan).
  - ○ Acute vasoplegic circulatory failure defined by treatment with vasopressors (adrenaline, noradrenaline, neosynephrine, vasopressin).
  - ○ Post-operative myocardial damage: Troponin I elevation of more than 10 times the 99th percentile (8)
  - ○ Myocardial infarction defined by an increase in cardiac enzymes (troponin I more than 10 times the 99th percentile) plus the appearance of a new Q wave, and/or ST segment elevation and/or a repolarisation disorder on the ECG and/or a de novo abnormality of the kinetics and/or an abnormality on coronary angiography.
  - ○ Ventricular rhythm disorders defined by the occurrence of ventricular tachycardia as evidenced by an electrocardioscopic recording / ECG.

- **Acute renal failure**, defined as an increase of at least 50% and/or 26.5 micromol/l in postoperative creatinemia compared with the preoperative basal value and/or diuresis of less than 0.5 ml/kg/h over 6 hours (KDIGO International Society of Nephrology definition) (71).

- **Digestive system** (68):
  - ○ Mesenteric ischaemia/ischaemic colitis documented by scannographic and/or colonoscopic and/or surgical imaging.
  - ○ Digestive haemorrhage (upper or lower) defined by the occurrence of haematemesis, melena or rectorrhagia. ○ Post-operative ileus defined by a cessation or temporary slowing of intestinal transit lasting more than 48 hours.

- **Haemorrhagic** :
  - ○ Postoperative bleeding defined by the Universal Definition of Perioperative Bleeding (UDPB) classification (72).
  - ○ Transfusion of blood derivatives: red blood cells, platelets, fresh plasma.

- **Infectious diseases** (73):
  - ○ Surgical site infection defined by the occurrence of a wall infection (abscess, purulent discharge) or defined by the occurrence of mediastinitis (scannographic imaging and bacterial documentation and/or surgical re-intervention).
  - ○ Symptomatic or asymptomatic postoperative urinary tract infection defined by the presence of a germ > 10^5 CFU/mm^3 in the urine.
  - ○ Pneumopathy defined by the appearance of new pulmonary infiltrates plus at least two of the following criteria: 1) fever > 38.5°C or hypothermia < 35.5°C; 2) leukopenia < 4,000 GB/mm^3 or hyperleukocytosis > 12,000 GB/mm^3 ; 3) purulent secretions. Bacteriological confirmation is based on the presence of micro-organisms in bronchial samples (ECBC > 107 CFU/mm^3 , bronchial aspirate > 10^5 CFU/mm^3 , BAL > 10^4 CFU/mm^3 , PDP or protected brush > 10^3 CFU/mm). 3
  - ○ Bacteremia defined by the presence of a blood culture positive for a pathogenic germ.
  - ○ Infection of a catheter (central venous, arterial) defined by evidence in culture of the same microorganism on the catheter (> 10^3 UFC ml^{-1}) and on at least 1 blood culture taken from the periphery.
  - ○ Sepsis defined by a SOFA Score ≥ 2 or increase of ≥ 2 points if organ dysfunction was present prior to infection. Specific arguments for infection include the presence of white blood cells in a normally sterile fluid (such as urine or cerebrospinal fluid), arguments for visceral perforation

(presence of air on an unprepared abdomen (UA) or abdominal/thoracic computed tomography (CT) scan, signs of acute peritonitis), presence of pneumonitis on a chest X-ray, petechiae, purpura or purpura fulminans.
  - Septic shock is defined as sepsis requiring vasopressors to maintain a mean arterial pressure of more than 65 mmHg associated with hyperlactatemia of more than 2 mmol L^{-1} despite the correction of hypovolaemia.

4 METHODOLOGY

4.1. Diagram of the study

This is a randomised, controlled, superiority, single-blind, two parallel-arm, multicentre clinical trial in patients undergoing cardiac surgery with bypass grafts.

Intervention arm: balanced general anaesthesia without opioid (OFA)
Control arm: standard balanced general anaesthesia (with opioid)

The study will be carried out in a single-blind fashion (the investigating doctor knows the allocated treatment, the patient does not) because it is comparing two different techniques that cannot be blinded. However, assessment of the clinical endpoints (post-operative complications) will be carried out in a blinded fashion by a nursing team from the intensive care unit in charge of the post-operative patient.

4.2 Calculating the number of subjects required

Assuming a post-operative complication rate of 60% in the control group (63), and taking into account adjustment for the stratification factors (centre and EUROSCORE 2), 288 patients (144 per group) will be required to demonstrate a 20% reduction in the complication rate in the OFA group, i.e. an OR of 0.444 (minimum expected difference taking into account the simultaneous consideration of several clinical events) with a power of 90% and a two-sided alpha risk of 5%. The software used for this estimate is PASS 11, and details of the procedure are available in appendix 3.
Anticipating 10% of patients for whom the primary endpoint cannot be assessed, 320 patients will be included.

4.3. Study population

4.3.1 Inclusion criteria

- Patient has given written, free and informed consent
- Patients over 18
- Patients scheduled for cardiac surgery
  a. Scheduled
  b. With CPB
  c. Types: aortic valve surgery, mitral valve surgery, tricuspid valve surgery, atrial myxoma, coronary artery bypass grafting, aortic surgery, combined surgery

4.3.2 Non-inclusion criteria

- Patient not affiliated to or not benefiting from a social security scheme
- Person under legal protection (curatorship, guardianship)
- Person subject to a legal protection measure
- Pregnant, parturient or breast-feeding women
- An adult who is incapable or unable to give consent
- Patient included once in the study
- Patients requiring emergency surgery within 24 hours
- Patients who are hypersensitive to local anaesthetics or opiates or to any of the excipients present in the products used.

- Patients taking antidepressants, neuroleptics such as non-selective MAOIs (iproniazid), selective A MAOIs (moclobemide), selective B MAOIs (selegiline), gabapentin (Neurontin®), etc.
- Patients with untreated atrioventricular conduction disorder
- Patient with prolonged QTc (> 450 ms) on preoperative ECG
- Patients with severe hepatic impairment (TP<30%)
- patients suffering from respiratory insufficiency (patients on long-term oxygen therapy, except those with OSA)
- Patients with uncontrolled epilepsy
- Patient with preoperative cognitive dysfunction (MMS <24)
- Patient with intracranial hypertension
- Patients with chronic renal failure (dialysis, creatinine > 200 µmol L) $^{-1}$
- Patients with porphyria
- Patient treated with linezolid (Zyvoxid®)
- Patients with severe arterial hypotension (SAP<90 mmHg)

4.3.3 Premature and definitive study exit criteria

A study withdrawal will be considered premature and definitive in one of the following cases: - Patient decision (withdrawal of consent)
- Lost patient
- Deaths
- Premature termination of research

The investigator should make every effort to contact patients lost to follow-up at D60, in order to obtain information about the patient at D30 (vital status, complications, etc.). Attempts to contact patients should be documented in the patient's medical record.

4.3.4 End of protocol study

Study exit corresponds to the date of the end of the protocol follow-up, i.e. 90 days after surgery (telephone call).

4.4 Project feasibility

The project is feasible for several reasons:

- Anaesthesia without opioid analgesia is used on a daily basis in the investigating centres for both cardiac and non-cardiac surgery. It is a modality performed and mastered by the various investigators.
- The research protocol does not change the usual practices of the investigating centres in any major way.
- The number of patients to be included is consistent and feasible, since Dijon University Hospital treats 900 patients a year, Strasbourg University Hospital around 1,200 patients a year, and Besançon University Hospital around 700 patients a year. Assuming that 10% of patients can be included, the study can be completed in two years.
- This research protocol is simple to implement and consistent.
- All the investigators have participated in previous comparable multicentre randomised trials (INPRESS, LICORNE, OPVI, PANEX, etc.).

4.5. Benefit-risk balance

The expected individual benefits are :

- An improvement in the overall peri-operative management of patients, with an improvement in post-operative analgesia and a reduction in the incidence of peri-operative complications,
- A reduction in opioid-related side-effects such as nausea and vomiting, post-operative cognitive dysfunction and bradypnea,
- An improvement in peri-operative cognitive function,
- A reduction in the length of stay in intensive care and in hospital.

This protocol is part of a global approach aimed at improving perioperative care, such as assisted recovery programmes after surgery. Cardiac surgery remains a high-risk surgery for which we need to improve our practices, because the incidence of complications, the length of hospital stay and the costs associated with these treatments are high. Reducing the incidence of perioperative complications and the length of stay in intensive care and in hospital would also represent a saving in terms of human and material resources and therefore a benefit in terms of public health.

The risks involved are systemic toxicity to local anaesthetics. However, it has been shown that blood concentrations using the protocol proposed in the study are around 2 µg ml^{-1} (Table 1), whereas signs of toxicity appear at higher plasma concentrations (6 to 10 µg ml^{-1}). This protocol is widely used and has been shown to be safe (no serious adverse effects). The retrospective study carried out in our centre found no increase in adverse events in the OFA group compared with the standard group. Several studies, including some carried out in cardiac surgery with CEC, confirm the safety profile of continuous intraoperative administration of lidocaine at a maximum dose of 3 mg Kg^{-1} h^{-1} (Table 1). In addition, there is an "antidote", **intralipid®,** which must be present and available in every operating theatre.

5. TREATMENTS

5.1. Experimental treatment

Opioid-free anaesthesia group (OFA) :

LIDOCAINE AGUETTANT 10 mg/ml SANS CONSERVATEUR, solution for injection. Marketing authorisation: 34009 362 725 4 5: 20 ml in bottle (glass). Box of 10.
ATC : N01BB02
Marketing authorisation: 24/10/1995
Marketing authorisation holder: AGUETTANT, 1 rue Alexander Fleming, 69007 LYON

Active substance: lidocaine hydrochloride anhydrous
A 20 ml vial contains 212.2 mg lidocaine hydrochloride monohydrate (equivalent to 200 mg lidocaine hydrochloride anhydrous).

Lidocaine causes a reversible loss of sensation by preventing or reducing the conduction of sensory nerve signals near their site of action; the primary site of action being the cell membrane. Lidocaine blocks conduction by reducing or preventing the significant and transient increase in sodium permeability of excitable membranes, a permeability normally produced by slight depolarisation of the membranes.

5.2. Treatment circuits

The treatment circuit for lidocaine hydrochloride is as follows.

5.2.1. Labelling of treatments

Commercial products from the stock of the coordinating PUI will be used. Labelling of the investigational medicinal product will be carried out by the coordinating internal-use pharmacy (PUI), in accordance with the decree of 24 May 2006, which specifies the content of labels for investigational medicinal products. The labels will be supplied by the sponsor.
The principal investigator in each centre or a pharmacist in each PUI will be responsible for keeping an inventory of products, checking expiry dates and storing medicines in the conditions recommended by the manufacturer.

5.2.2. Packaging and blinding of treatments (if applicable)

NA

5.2.3. Storage

The labelled treatment units will be stored by the PUI in a secure area with limited access, separate from the stock of non-investigational medicines and in accordance with the instructions given by the manufacturer.

Depending on the organisation of each centre, a limited quantity of treatment units may be supplied to the Anaesthesia and Intensive Care Department. In this case, the treatment units will be stored in the Anaesthesia and Intensive Care Department, in a secure area with limited access, separate from the stock of non-investigational medicinal products and in accordance with the instructions given by the manufacturer.

5.2.4. Dispensing treatments

Depending on the organisation of each centre, the treatment units will be dispensed by the PUI of each centre either by name for each patient, or according to an allocation in the Anaesthesia Intensive Care Department.

In the event of an endowment being set up, the treatment units will be taken from this endowment under the responsibility of the investigator for each patient.

Treatment dispensing will be :

- Nominative, on the day of the operation
- Notification of the randomisation arm will be sent to the investigator and the pharmacy.
- The prescription will be generated from the e-CRF and completed by the investigator
- The prescription will then be completed by the IADE for the administration part and faxed to the pharmacy for traceability.

5.2.5. Accounting and compliance

Insofar as administration is carried out in hospital by hospital staff, no compliance problems are expected. Nevertheless, the administration will be traceable by the IADE.
Treatment unit accounting will be monitored by the departments storing the treatment units, and a copy of the traceability documents will be sent to each centre's PUI.

5.2.6. Return and destruction of products

The treatment units used will be destroyed in each participating centre, after monitoring and approval by the promoter.
Unused treatment units will be returned to each centre's pharmacy for accounting and destruction after monitoring and approval by the sponsor.
Certificates of destruction will be drawn up by the authorised persons and sent to the promoter.

5.3. Associated treatments

The associated anaesthetic treatments are :

- Dexamethasone 0.1 mg Kg^{-1} intravenously
- Ketamine 0.5 mg Kg^{-1} intravenously
- Intravenous magnesium sulphate 30 mg Kg^{-1}
- Target concentrations of propofol are started at a target of 1.5 µg/ml and then increased in increments of 0.5 µg/ml to achieve a BIS of less than 60 (11).
- Cisatracurium (0.15 mg Kg^{-1}) or atracurium besilate intravenously with curameter monitoring
- Sufentanil 0.5 ng ml^{-1} (16).

The associated analgesic treatments are :

- Paracetamol administered intravenously (Perfalgan®) or orally (Dafalgan®) at a dose of 15 mg Kg^{-1} , 4 times a day.

- Morphine administered intravenously initially by a titration of 2-3 mg until VAS < 3, then self-controlled by the patient with the following parameters: bolus of 1 mg, refractory period of 7 minutes, maximum dose per 4 hours of 20 mg.

Emergency analgesia will be decided by the doctor when analgesia is insufficient (VAS >3) despite treatment with opioid (20 mg in titration or accumulated over the last 4 hours) and paracetamol, and will make available :

- Ketoprofen (Profenid®) 50 or 100 mg orally or intravenously for up to 48 hours
- Topalgic (Contramal®) 50 or 100 mg orally or intravenously

5.4. Unauthorised treatments

Locoregional analgesia (epidural analgesia, paravertebral block, serratus block, intercostal block, inter pectoral block) is not permitted in this study.

The prohibited treatments are :

- Gabapentin (Neurontin®)
- Nefopam (Acupan®) administered continuously intravenously
- Non-selective MAOIs (iproniazid)
- A-selective MAOIs (moclobemide)
- Selective B MAOIs (selegiline)

5.5. Management protocol

On arrival in the operating theatre

Placement of a peripheral intravenous catheter and infusion of lactated Ringer's solution at a rate of 1ml.kg.h^{-1} .

Patient monitoring with continuous electrocardiographic and phletysmographic surveillance.

Placement of a radial arterial catheter for continuous measurement of systolic and diastolic blood pressure, and a central venous catheter.

Continuous monitoring of the bispectral index (BIS). Placement of a urinary catheter to collect hourly diuresis.

Antibiotic prophylaxis according to the type of surgery, adapted from the recommendations of the French Society of Anaesthesia and Intensive Care (SFAR).

Protocol for induction and maintenance of general anaesthesia

1) The procedure for inducing general anaesthesia is standardised according to the group drawn.

Preoxygenation with spontaneous ventilation using a face mask with a FiO_2 of 100% (to obtain an expired fraction of O_2 > 90%). Anaesthetic induction by administration of opioid (control group) or lidocaine (OFA group), and a hypnotic (propofol) for a target of BIS between 40 and 60, and a curare (cisatracurium, tracrurium besilate) whose re-injections, if any, will be adapted to the train-of-four (TOF) response.

Intervention arm :

Anaesthetic *induction*

- Dexamethasone 0.1 mg Kg^{-1} intravenously
- Ketamine 0.5 mg Kg^{-1} intravenously
- Intravenous magnesium sulphate 30 mg Kg $^{-1}$
- Lidocaine 1.5 mg Kg-1 intravenously over 10 minutes
- Intravenous anaesthesia with a target propofol effect site concentration to achieve a BIS between 40-60, target propofol concentrations are started at a target of 1.5 µg/ml and then increased in increments of 0.5 µg/ml to achieve a BIS of less than 60 (11).
- Cisatracurium (0.15 mg Kg^{-1}), or atracurium besilate intravenously with curameter monitoring

Control group

Anaesthetic induction

- Intravenous anaesthesia with a target sufentanil concentration at the effect site of 0.5 ng ml[1] (16).
- Intravenous anaesthesia with a target propofol effect site concentration to achieve a BIS between 40-60, target propofol concentrations are started at a target of 1.5 µg/ml and then increased in increments of 0.5 µg/ml to achieve a BIS of less than 60 (11).
- Intravenous cisatracurium (0.15 mg Kg^{-1}) or atracurium with curameter monitoring.

2) After orotracheal intubation, patients are ventilated in volume-controlled mode. General anaesthesia is maintained by combining a hypnotic (propofol) in an air/oxygen mixture (BIS between 40 and 60) with the analgesic (sufentanil or lidocaine) used for anaesthetic induction.

Intervention arm :

Maintenance

- Continuous infusion of intravenous lidocaine, using a syringe pump, at a dose of 1.5 $mg.Kg^{-1}.h^{-1}$ until sternotomy, then 1 $mg.Kg^{-1}$ $.h^{-1}$ until it stops at surgical closure.
- Intravenous anaesthesia with a target concentration at the propofol effect site to obtain a BIS between 40-60, target propofol concentrations are increased in steps of 0.5 µg/ml and decreased in steps of 0.2 µg/ml without falling below 1 µg/ml.

Control group :

Maintenance

- Intravenous anaesthesia with a target sufentanil concentration at the effect site of between 0.5 and sternotomy, then reduced to 0.3 ng ml^{-1} , stopped at the end of the procedure (16).
- Intravenous anaesthesia with a target concentration at the propofol effect site to obtain a BIS between 40-60, target propofol concentrations are increased in steps of 0.5 µg/ml and decreased in steps of 0.2 µg/ml without falling below 1 µg/ml (16).

In the 2 study groups, the use of locoregional analgesia was not authorised during the operative period.

Mechanical ventilation management

All patients are intubated and ventilated in controlled mode with a tidal volume (Vt) of between 6 and 8 ml Kg^{-1} of ideal theoretical weight, a respiratory frequency adapted to maintain an $EtCO_2$ of between 30 and 35 mmHg and $PaCO_2$, an I/E ratio of 1/2 and a positive end-expiratory pressure of 5 to 10 cm H_2 O. Recruitment manoeuvres are performed regularly (at least every 60 minutes). These consist of applying a continuous positive end-expiratory pressure over a limited period of time. The inspired fraction of oxygen is adjusted to obtain an SpO_2 of between 95 and 97%.

Strategy for optimising cardiac preload

All patients are monitored using a tool for continuous measurement of cardiac output (trans-oesophageal cardiac ultrasound, Swan-Ganz, pulse wave contour analysis). Haemodynamic optimisation is based on continuous measurement of ESV in accordance with SFAR recommendations.

Blood loss will be compensated for by transfusion of packed red blood cells on the basis of intraoperative haemoglobin monitoring by spectrophotometry, as usually performed.

Blood pressure management strategy

The objective of maintaining blood pressure is based on the measurement of blood pressure. This objective is achieved according to the following usual procedures.

The usual management of arterial hypotension is the administration of ephedrine hydrochloride (0.15 mg kg^{-1} IVD) in increments until MAP is corrected. If the episode of arterial hypotension is not corrected by ephedrine hydrochloride (after a maximum of 60 mg), noradrenaline may be administered. The dosage of noradrenaline depends on the individual. The initial infusion rate is started at a mean dose of 0.05 µg/kg/min; the infusion rate is titrated to maintain MAP targets.

The products are administered in accordance with the recommendations for clinical practice (Appendix).

- Ephedrine hydrochloride (3 mg ml $solution^{-1}$): administration by repeated intravenous boluses when arterial hypotension (as defined above) is detected. Initial bolus of 6 mg, followed by possible reinjections of 3 mg until target blood pressure is restored, up to a maximum of 60 mg. If treatment is ineffective, noradrenaline may be used.
- Noradrenaline tartrate (10 µg ml $solution^{-1}$): administration by continuous intravenous infusion using an electric syringe on a dedicated peripheral venous line. The product is diluted to a concentration of 100 µg ml^{-1} by the anaesthetist or nurse in charge of the patient on arrival in the operating theatre. The initial infusion rate is 0.05 µg kg^{-1} min^{-1} . The infusion rate is adjusted to maintain MAP targets.

In both groups, bradycardia was defined as a heart rate below 40 bpm. It was systematically treated with 0.1 mg kg^{-1} of atropine.

- If arterial hypertension WITHOUT sinus tachycardia: titration with URAPIDIL 5 mg intravenously.
- If arterial hypertension AND sinus tachycardia (heart rate > 85 BPM): titration with intravenous ESMOLOL in boluses of 5 to 10 mg repeatedly.

Analgesia management strategy

Intra-operatively

Intraoperative analgesia initiated 30 minutes before the end of the operation

- Intravenous paracetamol (Perfalgan®) at a dose of 15mg/kg^{-1} IVL - Intravenous opioid at a dose of 3mg bolus IVD (2mg if patient's age > 75).

No loco-regional analgesia is authorised.

Post-operative

The associated analgesic treatments are :

- Paracetamol administered intravenously (Perfalgan®) or orally (Dafalgan®) at a dose of 15 mg Kg^{-1} , 4 times a day.
- Opioid administered intravenously initially by a titration of 2-3 mg until VAS < 3 then self-controlled by the patient with the following parameters: bolus of 1 mg, refractory period of 7 minutes, maximum dose per 4 hours of 20 mg. **No loco-regional analgesia is permitted.**

6. progress of the study

6.1. Pre-selection of patients

Recruitment is carried out during the anaesthesia consultation by the anaesthetist-intensive care physician on the premises of the anaesthesia-intensive care department or on the ward where the patient is hospitalised if he or she is unable to attend.

The anaesthetic consultation is carried out between 2 months and 48 hours before the operation. The patient will then be offered the opportunity to take part in the study, and will be given an information note to help them make their decision. This consultation is compulsory before any operation involving general anaesthetic. However, signed consent

will only be obtained during the pre-anaesthetic visit on the day before or the day of the operation, to allow the patient time to reflect.

6.2 Information and obtaining consent

If the patient meets the eligibility criteria, the investigating doctor will present the study. He will inform the patient about the nature of the research, its objectives, methodology, duration, expected benefits, constraints and foreseeable risks, in accordance with article L1122-1 of the CSP.
If the patient agrees, two copies of the written, free and informed consent form will be collected by one of the investigating physicians. One copy of the information form and the signed consent will be given to the subject, and the other original copy of the consent will be kept by the investigator. The investigator must record in each patient's medical file the fact that the patient has been informed and is included in a research protocol.
The patient will then be included after verification of the inclusion and non-inclusion criteria.
All patients taking part in the study must undergo a preliminary medical examination adapted to the research, which is the one carried out during the anaesthetic consultation: clinical examination including co-morbidities and treatments.

6.3 Inclusion procedures

The pre-anaesthetic visit is carried out the day before the operation or on the same day, and is used to ensure that the patient's state of health has not changed since the anaesthetic consultation and that the patient still meets the criteria for inclusion in the study. Signed consent will be obtained at this visit.
In addition, data will be collected during the standard examination: Demographic criteria, Preoperative clinical and drug criteria, Surgical criteria

6.4. Randomisation procedures

Randomisation will be carried out centrally on the day of the intervention, using software directly accessible online by the investigators (CleanWeb software), after identification using a password. Compliance of the data with the inclusion and non-inclusion criteria will be checked at the time of the randomisation request.

Allocation: The treatment allocation algorithm will be established by the statistician of the Research Methodology Support Unit (USMR) of the Dijon DRCI. Patients will be randomised using a minimisation technique with stratification based on Euroscore 2 score (< 3, ≥ 3) and inclusion centre. Two groups will be identified: "Control" and "OFA". The 2 treatment groups are balanced with a 1:1 ratio. A full document describing the randomisation procedure is kept confidential at the USMR.
In the event of difficulties during randomisation, the investigator may contact **the Research Methodology Support Unit (USMR) of the DRCI, Dijon Bourgogne University Hospital.**

Blind: The study is single-blind because it compares two different techniques that cannot be blinded.
Only the anaesthetist and the nurse anaesthetist in charge of the patient during the operation will be informed of the assignment group; they will not be involved at any time in the post-operative management of patients. Post-operatively, patients and the medical and paramedical team will be kept blind to the randomisation group throughout the hospital stay following surgery.

6.5. Monitoring procedures / questionnaires

The clinical evaluation and biological tests are those usually carried out for patients undergoing cardiac surgery. No additional clinical or paraclinical examinations are required.

These parameters are :
- Clinical: blood pressure, PVC, heart rate, temperature, diuresis

- Biologicals for all patients included: blood count, haemostasis, blood ionogram, urea, creatinine, CRP, PCT, BNP, capillary glycaemia, blood glucose, albumin, arterial and venous blood gases, troponin, liver function tests, CK, CKMB, LDH
- Imaging: cardiac ultrasound, chest X-ray, renal and pulmonary ultrasound

Patients will be monitored on a daily basis in the intensive care unit until 7 days after surgery, or until discharge from hospital if this takes place before 7 days:
- Data for the primary and secondary endpoints will be collected by a clinical study technician. These data will be collected in an observation notebook in accordance with the recommendations. - Pain assessment (visual analogue scale at rest and when coughing) will be collected from the monitoring sheet at 48 hours after extubation.
- Daily morphine consumption will be recorded on the monitoring sheet on D1 and D2 of the surgery.
- The occurrence of nausea and vomiting will be recorded on the nurses' transmission sheet following the first 48 hours after extubation.
- In accordance with the service protocols for this type of surgery, a standard biological work-up for

all patients included are sampled on admission to the intensive care unit and on D1 and D2 of surgery
- A quality of recovery score after surgery (QoR15) will be carried out on discharge from the intensive care unit.
- Collection of drug treatments administered

A follow-up consultation is held with the surgeon between D30 and D60 after surgery. During this consultation, the following will be recorded: late complications (between discharge from hospital and D30) and the total length of stay in intensive care and in hospital.

If the consultation could not take place, a telephone call at D60 (+/- 7 days) would be made to assess these elements and the vital status (the information gathered should correspond to the patient's condition at D30).

6.6. For patients included at the CHU Dijon Bourgogne :

- Additional samples taken during routine care:
- a lipid profile
- determination of LPS by mass spectrometry
- LPS activity in LAL
- fluorometric assay of phospholipid transfer protein (PLTP) activity
- measurement of cytokines, CRP and PCT - measurement of I-FABP, GLP-1, Citrulline and Zonulin. At given times :
- Before surgery
- At the end of the ECC
- 4 hours after CEC
- Day 1 after surgery
- 2 days after surgery

An EDTA tube (4 ml) is taken at different times. It is centrifuged to separate the pellet from the plasma. The plasma is recovered and aliquoted and stored at -80°C in the UMR1231 laboratory freezer. Samples are identified by an anonymous code based on the randomisation number and the sampling time.

6.7. End of the study

The patient left the study at D90 (+/- 7 days) following a telephone call to assess vital status since the last visit.

6.8. Technical description of measured parameters

The clinical assessment and biological tests are those usually carried out for patients undergoing cardiac surgery.

6.9. Description of permanent or temporary shutdown rules

A person's participation in research :

All those included will be followed until the end of the study, except for patients who have withdrawn their consent.

All or part of the research (if applicable)
The trial may be stopped at any time in the event of systemic toxicity to local anaesthetics: unexpected frequency and/or severity of toxicity.
The investigator may also terminate the trial if too few patients are included in the study within the planned timeframe.

6.10. Period of exclusion - exclusivity

**Exclusivity period**<br>If yes, the patient may not participate simultaneously in other research	yes ☒<br>no ☐
**Exclusion period**<br>If yes, the patient may not take part in any other research after this one has ended Duration: .... this for a defined period	yes ☐<br>no ☒

The patient cannot take part in other research evaluating surgical techniques.

6.11. Duration of the study

Duration of inclusion	30 months
Duration of participation for one person	90 days
Total duration of study (from 1st inclusion to last follow-up of last patient)	33 months

Protocol: OFACAR Version: 3Date: 04/01/2022

6.12. Study organisation chart

	Screening	Randomisation	EXTUBATION	Data collection							End of
	J-x	D0: Surgery		Extubation	H+48	J1	J2	J3	J4, J5, J7	Between D6	J90 call (+/- 7 day
Anaesthesia consultation	J-x										
Clinical evaluation	J-x										
Patient information	J-x										
Informed consent	J-1										
Cardiac		x									

**Primary endpoint**				x	x	x	x	x	**X to D30**	
**Biological**					x	x				
**Additional tubes for Dijon patients**		- Before surgery<br>- At the end of the ECC			x	x				
**EVA pain at rest and on**				x						
**QoR15 questionnaire**					**On leaving the intensive care unit**					
**Complications**				x	x	x	x	x	**X to D30**	
**Vital status**				x	x	x	x	x	**X to D30**	x
**Nausea,**				x						
**Length of stay in intensive**								x	x	
**Serious adverse**		x		x	x	x	x	x	**x (up**	
**Dispensing treatment**		x								

Protocole RIPH - trame française /DRCI-PPC-ELA14045.3/ Created Propriété du CHU Dijon Bourgogne Page 33 sur 57 le : 29/11/2017 - Modified on : 12/04/2019

7. DATA MANAGEMENT

7.1. Confidentiality

Only the patient's code is recorded in the CRF. This consists of the patient initials (first letter of the surname and first letter of the first name), the centre number and the inclusion rank number. When the data is extracted, only the rank number is retained.
Ownership and use of the data will be exclusive to the Study Sponsor.

7.2. Collection of data

The data will be collated in an electronic CRF (e-CRF) created from CleanWeb software by a data manager. Data entry will be directly accessible online at the following address after logging in with a login and password:
https://chu-dijon.tentelemed.com/Ctms-chud/portal/login

Demographic criteria :

- Weight
- Size
- Age
- Gender
- ASA score (1, 2, 3, 4)
- Euroscore 2 (additive, logistics)
- Apfel score
- Comorbidities: hypertension, insulin-dependent diabetes, non-insulin-dependent diabetes, dyslipidaemia, obliterating arterial disease of the lower limbs (AOMI), aortic aneurysm, carotid stenosis (> 50%), chronic renal failure, coronary artery disease, hypothyroidism, systemic disease (lupus, etc.), stroke/TIA, PAH, chronic respiratory failure (COPD) on oxygen therapy, sleep apnoea syndrome, etc.
- Ventricular aneurysm
- Preoperative FeVG
- Preoperative creatinine

Preoperative drug criteria: yes/no

- Beta-blockers yes/no, if yes: taken on the morning of the operation yes/no
- Calcium inhibitor
- IEC: yes/no, if yes stop 24 h before (date)
- ARA $_2$
- Digitalique
- Amiodarone
- Sotalol
- Neprilysin inhibitors
- Aspirin
- Clopidogrel
- Brilique
- New oral anticoagulant (xarelto, pradaxa)
- LMWH/HNF
- VKA
- Statin
- Loop diuretic
- Thiazide
- Insulin
- Hypoglycaemic sulphonamide
- New oral antidiabetic
- Levothyrox
- Corticosteroid therapy
- Antibiotic therapy

Surgical criterion :

- Type of surgery: valve (mitral, aortic, tricupsidial, pulmonary, mixed, biological, mechanical, plastic), coronary artery bypass graft (number), combined (valve and bypass graft), ascending aorta, aortic arch, other (myxoma, AIC closure, thromboendarterectomy, etc.).

Randomisation :
- Type of anaesthesia: OFA or control

Operating parameters :
- Hypertensive episodes PAS > 140 mmHg (yes/no)
- Hypotensive episodes PAS < 95 mmHg (yes/no)
- Episodes of bradycardia HR < 45 (yes/no)
- Episodes of tachycardia HR > 90 (yes/no)
- CEC duration (minutes)
- Duration of aortic clamping (minutes)
- Duration of anaesthesia (minutes)
- Biological check-up for Dijon patients at the end of bypass surgery
- Electro systolic entrainment (EES) (yes/no)
- Total dose of propofol (mg)
- Total dose of sufentanil (mg)
- Total dose of lidocaine (mg)
- Total dose of ketamine (mg)
- Total heparin (IU)
- Protamine (IU)
- Capillary blood glucose (mmol l) after anaesthetic induction, before bypass surgery, during bypass surgery, before return to intensive care
- Total dose of insulin in the operating theatre (IU)
- Total electrolyte intake (ml, crystalloids/colloids)
- Transfusion of blood derivatives (type: PFS, platelets, overall and quantity: number of bags)
- Tranexamic acid (mg) - Catecholamines :
  - Ephedrine (total dose mg)
  - Phenylephrine (total dose µg bolus)
  - Noradrenaline (duration, maximum dosage: maximum perfusion rate)
  - Dobutamine (duration, maximum dosage: maximum perfusion rate)
  - Adrenaline (duration, maximum dosage: maximum perfusion rate)
  - Phosphodiesterase inhibitor (duration, maximum dosage)
  - Levosimendan (duration, maximum dosage)
- Total diuresis per operation (ml)
- ECLS (yes/no)
- Intra-aortic counterpulsation balloon (BCPIA) (yes/no)

D0: surgery - D1 and D2:
- Extubation (yes/no if yes, date and time)
- Complications (type, number)
- SOFA score
- EES (Electro Systolic Training - Pace Maker) (yes/no) - Arterial lactates - Catecholamines :
  - Noradrenaline (duration, maximum dosage: maximum infusion rate)
  - Vasopressin (duration, maximum dosage: maximum infusion rate)
  - Dobutamine (duration, maximum dosage: maximum infusion rate)
  - Adrenaline (duration, maximum dosage: maximum infusion rate)
  - Phosphodiesterase inhibitor (duration, maximum dosage)
  - Levosimendan (duration, maximum dosage)
- Total dose of insulin received (IU)

- Collection of the number of VAS for pain at rest and coughing during the 48 hours after extubation
- Collection of number of episodes of nausea-vomiting in the 48 hours after extubation
- Standard biological work-up on D1 and D2 of surgery
- Additional analgesia (yes/no, if yes type)

On discharge from the intensive care unit :

- QoR15 questionnaire
- Total dose of morphine received (mg)

-

From D3 to D7

- Complications (type, number)

At the post-surgery consultation (carried out between D30-J60)

- Complications (type, number) between D7 and D30
- Total length of stay in hospital and intensive care unit
- Serious and non-serious adverse events between D7 and D30

If this consultation has not taken place, a telephone call will be made at D60 (+/- 7 days) to collect the above information. Please note that this information must correspond to the patient's condition at D30.

During the telephone call at D90 (+/- 7 days)

- Vital status

7.3. Quality and Safety Manager

Safety, quality and access to study data will be managed by the USMR team at the CHU DIJON BOURGOGNE.

Data entry will be validated live by dynamic consistency checks implemented during database construction.

A series of queries will be carried out periodically during the project by a data manager, in conjunction with the monitoring visits, with the aim of validating the consistency of all the data. These queries will be repeated iteratively until an error-free database is obtained.
Missing or inconsistent data will be checked regularly and queries will be sent to the investigators by the study data manager in order to minimise their impact on the analysis.

7.4. Storage and archiving

The CleanWEB software is hosted by Telemedicine Technologies via its secure Internet hosting platform.

A copy of the extraction file in "csv" format from the frozen database will also be stored on a Dijon Bourgogne CHU server, secured by a password by the data manager.

8. COORDINATION OF THE STUDY

8.1. Steering committee

The steering committee is responsible for steering the trial from conception to publication.

The committee is made up of the co-ordinating investigator (Pr B. Bouhemad), the principal investigator in Dijon (Pr PG. Guinot), an associate investigator in Dijon (Dr N. Nowobilski), the study methodologist (Dr A. Soudry-Faure), a Clinical Study Co-ordinator (USMR de la
DRCI) and a representative of the study sponsor (CHU Dijon Bourgogne)

The committee will meet every 6 months until the end of the monitoring period.

8.2. Independent Supervisory Committee

The Independent Monitoring Committee (IMC) is an independent advisory committee. ISC meetings will provide an opportunity to review the safety of the trial. This review will be based on 1) the description of the AEs and MAEs collected (severity, frequency and consequences), 2) the progress of recruitment and 3) the results of the analyses.

Reasons for setting up: Phase III drug trial, study population at risk.

The committee will be made up of at least one clinician specialising in anaesthesia and intensive care, one pharmacovigilant and one methodologist/statistician.

The practical details of how the committee operates are set out in a charter, which will be amended in line with the committee's requests at meetings.

The project manager, representing the promoter, has an informative and logistical role. He transfers information to the members of the ISC, organises the meetings and draws up the minutes. He does not take part in debates or votes.

9. SAFETY ASSESSMENT

9.1. Description of safety assessment parameters

Safety will be assessed by evaluating the general, clinical and biological condition of patients, and recording any serious adverse events between inclusion and D30.

9.2 Adverse events and reactions, definitions

9.2.1. Definitions

Adverse event: any noxious occurrence in a person undergoing research involving the human body, whether or not this occurrence is related to the research or to the product to which the research relates.

Adverse reaction to an investigational medicinal product: any noxious and undesirable reaction to a medicinal product, regardless of the dose administered.

Serious adverse event or reaction: any adverse event or reaction which:
- results in death,
- endangers the life of the person undergoing the research,
- requires hospitalisation or prolongation of hospitalisation,
- causes significant or lasting disability or handicap,
- results in a congenital anomaly or malformation, foetal malformation or abortion,
- is considered a significant medical event.

An "important medical event" is considered to be any event requiring intervention to prevent an outcome corresponding to one of the aforementioned seriousness criteria or any other medically important event as judged by the investigator.

Severity: The severity of events is estimated according to the NCI-CTCAE version 5.0 classification ranging from grade 1 to grade 5.

Unexpected adverse reaction: any undesirable effect of the product whose nature, severity, frequency or course are not consistent with the reference safety information.

Please note that the safety reference information may change during the course of a clinical trial. The version that applies is that in force at the time of the serious adverse reaction, including follow-up reports.

New event: any new data which may lead to a reassessment of the risk-benefit balance of the research or of the product which is the subject of the research, to changes in the use of this product, in the conduct of the research, or in the documents relating to the research, or to the suspension, interruption or modification of the research protocol or similar research. For trials involving the first administration or use of a health product in people with no medical condition: any serious adverse reaction.

Urgent safety measure: measure consisting of stopping the trial or taking immediate measures, implemented by the sponsor and/or investigator when a new fact is likely to affect the safety of the persons taking part in the clinical trial, in order to protect them against an immediate danger.

9.2.2. Research events

In this trial, non-serious adverse events are not specifically collected. Instead, the investigator collects intraoperative and postoperative complications as described in the objective and data collection sections of the protocol.
Serious adverse events are collected between D0 and D30, and without any time limit if they are judged to be reasonably related to the protocol treatment.

In the context of this trial, the following serious adverse events do not require immediate notification to the sponsor:
- an event leading to a transient move to a hospital consultation, door service or day hospital,
- hospitalisation (more than one night on site) or extended hospitalisation for the following reasons: ○ scheduled hospitalisation for routine procedures or treatments as part of a pre-defined monitoring or therapy programme,
    - hospitalisation or intervention required by the protocol,
    - hospitalisation for exploration not linked to a change in the patient's condition ○ hospitalisation for comfort or social reasons (e.g. hospitalisation of an elderly person who is dependent on the spouse who has just been hospitalised)
    - elective hospitalisation not associated with a worsening of the clinical condition and not related to the objective of the clinical study and taking place during the clinical study (e.g. cosmetic surgery),
    - any event linked to the progression of the disease

In this trial, the investigational medicinal product is lidocaine. The reference document is section 4.8 of the Summary of Product Characteristics of LIDOCAINE AGUETTANT preservative-free 10 mg/ml sol inj®.

9.2.3. Responsibilities of the investigator

The investigator records all complications in the observation book, as they become known.
The investigator notifies all serious adverse events without delay from the day on which he becomes aware of them. This notification is the subject of a written report and is followed by further detailed written reports.

The completed serious adverse event report form should be sent by fax to the DRCI of the CHU DIJON BOURGOGNE on 03.80.29.36.90. If it is not possible to send the report by fax, it can be sent by e-mail to the following address: **vigilance.ec@chu-dijon.fr.**

Additional information may be requested by the sponsor. The investigator must ensure that his report is documented as accurately as possible. To this end, they will provide all the documents required to analyse the case as soon as possible: hospitalisation report, imaging report, results of biological tests, etc. These documents will be made anonymous.

If an adverse event consists of several signs or symptoms that can be represented by a single syndrome or diagnosis, **it is preferable to include this syndrome or diagnosis on the report.**

The investigator is responsible for the appropriate medical follow-up of patients until resolution or stabilisation of the event or until the patient's death. This may sometimes mean that follow-up continues after the patient has left the study.

The investigator must send additional information and follow-up on the serious adverse event to the sponsor without delay after obtaining it. This should be done using the serious adverse event report form, specifying that it is a follow-up report (tick box). They also send the promoter the last follow-up report on the resolution, stabilisation or death of the patient.

If pregnancy is discovered after inclusion, the patient must be excluded from the study. The sponsor should be informed immediately using the Serious Adverse Event Form (no severity criteria should be ticked). The patient should be followed until the outcome of the pregnancy and this outcome, whatever it may be, should be reported to the sponsor.

9.2.4. Role of the promoter

In accordance with Article R1123-53 of the French Public Health Code and its implementing decrees and in compliance with the ANSM decision of 26 December 2016 setting the form, content and procedures for adverse reaction and new event reports, the sponsor shall report to the competent authority any suspected serious unexpected adverse reaction occurring in France and outside national territory within the following timeframes:
1.in the event of a serious unexpected adverse reaction resulting in death or which is life-threatening, without delay from the day on which the sponsor becomes aware of it,
2.in all other cases, at the latest within 15 days of the day on which the promoter becomes aware of it,
In the first case, the promoter declares the relevant additional information in the form of a follow-up report within 8 days of the initial declaration made by the promoter. In other cases, the sponsor must declare this information within 8 days of the deadline mentioned in situation 2.

The initial declaration without delay means that the promoter is aware of the following minimum information:
- a suspect experimental drug
- a person who has experienced the adverse reaction, identifiable in particular by his/her identification code number in the research concerned,
- a suspected serious and unexpected adverse reaction
- an assessment of causality
- an investigator or any other identifiable notifier
- a unique research identifier or protocol number allocated by the sponsor

In accordance with Article R1123-59 of the French Public Health Code and its implementing decree and in compliance with the ANSM decision of 26 December 2016 setting the form, content and procedures for adverse event and new

development reports, the sponsor shall inform the competent authority and the personal protection committee of new developments without delay.

10 STATISTICAL ANALYSES

10.1 Analysis population

The main analysis will be carried out on an intention-to-treat basis: all patients included in the trial and randomised will be taken into account.

The analysis will be completed by a per-protocol analysis taking into account the patients included who complied fully with the trial protocol instructions. Patients will be excluded if they have received a dose different from that provided for in the protocol, or if there was an error in randomisation or in the inclusion/exclusion criteria, etc. The per-protocol analysis will use the same techniques as the intention-to-treat analysis.

The main conclusion of the trial will relate to the intention-to-treat analysis only.

10.2 Choice of statistical tests

Descriptive analysis

To describe the characteristics of the population, quantitative variables will be described using the mean ± standard deviation and the median [Q1; Q3]. Qualitative variables will be described by their number (%). The characteristics of the two groups will be compared using the usual univariate tests (chi-2 test or Fisher's exact test for qualitative variables, Student's or Wilcoxon's test for quantitative variables), depending on the conditions of application.

Analysis of primary endpoint:

The effect of the type of anaesthesia (treatment groups) on the rate of occurrence of postoperative complications will be analysed by logistic regression adjusted on the stratification factors (centre and Euroscore 2).

If factors at inclusion appear to be unbalanced between groups, a multivariate logistic regression including these factors in addition to the stratification factors will be carried out in a second phase, making it possible to check the robustness of the conclusions of the main analysis.

Analysis of secondary endpoints :

Quantitative variables will be compared between the 2 arms using an ANOVA adjusted for stratification factors or the Wilcoxon test, depending on the distribution of the variables.
Categorical variables were compared between the 2 arms using logistic regression adjusted for stratification factors.

The time to extubation will be described and compared between the two groups using Kaplan-Meier curves and a logrank test if the application conditions are met.

As with the main analysis, in the event of unbalanced factors at inclusion, multivariate analyses including these factors in addition to the stratification factors will be performed.

Intermediate analyses :

The safety of the trial (rate of SAEs between inclusion and D30) and the rate of occurrence of post-operative complications (PJC) will be described and analysed according to the treatment arm after inclusion of 25% and 50% of patients. These results will be submitted to the ISC, which will decide whether the trial should be stopped. Only the ISC has access to the comparative results of the study data by

treatment arm during the course of the study. The significance threshold for these analyses will be lowered so as not to impact the main analysis (see 10.5).

10.3 Analysis of missing data:

For statistical analyses, the choice of treatment for missing data will depend on the variables concerned, the structure and the quantity of missing data.
The profile of missing data (rates by variable, by patient, by structure (missing completely at random (MCAR), missing at random (MAR), missing not at random (MNAR)) will be described. Depending on the results of this description, the decision to impute or not will be made. If the rate of missing data is less than 5%, then the impact will be deemed negligible and imputation will not be carried out. If the rate of missing data is between 5% and 30% and their structure allows it, an imputation will be carried out. If more than 30% of data is missing, no imputation will be carried out.

In the case of imputation, the method used will depend on the structure of the missing data. If they are missing completely at random (MCAR), a simple imputation method (by median, mean, regression, etc.) may be considered. Otherwise, the multiple imputation method will be preferred: this iterative method aims to obtain several imputed bases using a data set defined by all the variables useful for the analysis, then to carry out the analysis on all these bases and finally to pool the estimates to obtain the final results. Mathematical methods not detailed here are applied at each stage, their choice depending, among other things, on the quantitative or qualitative nature of the variables imputed and analysed.

If the data is imputed, a sensitivity analysis will be carried out on the complete cases to check the stability of the estimates obtained.

10.4 Choice of analysis software

SAS software version 9.4

10.5 Significance threshold

A significance level of 5% will be used for all final analyses.
For intermediate analyses: an alpha risk of 0.001 is used for each safety analysis of the trial (according to the method proposed by Peto-Haybittle so as not to modify the significance threshold of the final main analysis).

10.6 Person responsible for the analysis and place of analysis

The analyses will be carried out by the DRCI Methodological Research Support Unit team at Dijon Bourgogne University Hospital under the responsibility of Dr A Soudry-Faure in collaboration with Pr Belaid Bouhemad, Anaesthesia and Intensive Care Department.

11 ethical and regulatory aspects

11.1 Ethical conduct of the study

The planning and conduct of this study are governed by French and European laws (Law no. 2012-300 of 5 March 2012 on research involving the human person, amended by Order no. 2016-800 of 16 June 2016 and its implementing decrees). This research may only begin once all the legal provisions relating to obligations prior to the implementation of research have been complied with. The

study will be conducted in accordance with the ethical principles of the Declaration of Helsinki and the recommendations of Good Clinical Practice.

In accordance with article L1121-1 of the French Public Health Code, this study constitutes *category 1* research involving the human person, in that *it* constitutes ***interventional research involving an intervention on the person that is not justified by his or her usual care.***

11.2 Responsibilities of the investigator

• The investigator undertakes that this study will be carried out in accordance with Law no. 2012-300 of 5 March 2012 relating to research involving the human person, as amended by Order no. 2016-800 of 16 June 2016 and their implementing decrees, the Declaration of Helsinki and Good Clinical Practice. All data, documents and reports may be the subject of audits and regulatory inspections, without medical confidentiality being invoked.

• The investigator will inform the volunteers of the objectives and constraints of the study, and of their right to refuse to take part in the study or to leave it at any time. Once the subject has been informed and the investigator has ensured that he/she has fully understood the implications of participating in the study, his/her written consent will be collected by one of the investigators in duplicate. One copy of the information form and the signed consent will be given to the subject, and the other original copy of the consent will be kept by the investigator.

The consent process will be documented in the medical record.

• All information collected is confidential and may not be divulged. The investigator will ensure that the anonymity of each volunteer participating in the study is guaranteed. No information allowing the identification of individuals will be communicated to third parties other than those, representing the sponsor and the Ministry of Health, who are legally authorised to hold this information (and who are bound by professional secrecy).

11.3 Responsibilities of the promoter

The DIJON BOURGOGNE University Hospital is promoting this study.

In accordance with Law No. 2012-300 of 5 March 2012 on research involving the human person, amended by Order No. 2016-800 of 16 June 2016 and their implementing decrees, the sponsor undertakes to carry out all the operations for which it is responsible:

- **Registration of the study** at European level (EUDRACT no.).
- **Information or request for authorisation from the French National Agency for the Safety of Medicines and Health Products (ANSM**)
- **Submission to the Comité de Protection des Personnes**.
- **Declaration or request for authorisation from the Commission Nationale Informatique et Liberté**.
- **Insurance cover** for interventional research as defined in 1° and 2° of Article L1121-1 of the Public Health Code.
- **Substantial amendment:** After the study has begun, any substantial amendment to the protocol initiated by the investigator must be submitted to the sponsor, who must obtain a favourable opinion from the CPP and/or authorisation from the competent authority before implementing it.

11.3.1 Personal Data Protection Committee and Competent Authority

The study cannot begin without the dual authorisation of a Committee for the Protection of Individuals and the Competent Authority.

The protocol was approved by the CPP Sud Est VI on *23/03/2021* and authorised by the Agence Nationale de Sécurité du Médicament et des Produits de Santé on *17/03/2021.*

The competent authority's **authorisation lapses** if, **within two years** of authorisation, the research has not begun (i.e. no person included in the protocol). **The authorisation of** the Committee for the Protection of Individuals **lapses** if, **within two years** of the favourable opinion, the research has not begun (i.e. no person included in the protocol).

Neither the investigator nor the sponsor may modify this protocol without the prior written agreement of the other party. If substantial changes are to be made, these must be set out in an amendment to the protocol.
This amendment will be applied once it has received the dual authorisation of the CPP and/or the authorised Competent Authority.

11.3.2 Protection of personal data

The computer file used to carry out this research will be subject to a commitment of compliance with the C.N.I.L. pursuant to the "informatique et liberté" law, *law no. 78-17 of 6 January 1978 relating to information technology, files and freedoms, as amended, and to the General Regulation on the Protection of Personal Data (RGPD), adopted at European level, and which came into force on 25 May 2018*.

11.3.2.1 CNIL

The processing of the information gathered during this study will be done in accordance with the MR001 reference methodology. Declaration no. 2210226 v 0 dated 03 December 2018.

11.3.2.2 Confidentiality

In accordance with the provisions of article R5121-13 of the French Public Health Code, the investigator and any person called upon to collaborate in the studies are bound by professional secrecy, in particular with regard to the nature of the products studied, the studies, the persons involved and the results obtained, subject to the provisions of article L1123-14 of the French Public Health Code.

Without the agreement of the sponsor (Dijon Bourgogne University Hospital), they may only provide information about the study to the Health Authorities, including the inspectors as mentioned in article R5121-13 of the Public Health Code.

The studies will not be the subject of any comment, oral or written, without the joint authorisation of the coordinating investigator and the sponsor (Dijon Bourgogne University Hospital).

Medical data concerning patients will only be transmitted to the sponsor and, where applicable, to the authorised health authorities, under conditions which guarantee confidentiality. Patients may exercise their rights of access and rectification by contacting their investigator.

11.3.3 Insurance/Compensation for damage to patients

The sponsor has taken out insurance to cover its civil liability in the event of any harmful consequences arising in the course of this research.
In accordance with current legislation (article L. 1121-10 of the French Public Health Code), each patient is insured against any deterioration in his or her state of health that may result from participation in the study. The insurance company of the CHU Dijon Bourgogne is Société Hospitalière d'Assurances Mutuelles (SHAM) Policy no. 129.234.
The investigator must immediately report to the Clinical Research Department of the Dijon Bourgogne University Hospital any complaint made by a patient which

may be related to the study. The Clinical Research Director will forward this complaint to the Legal Department.

11.3.4 Authorisation, persons responsible and study sites

An Authorisation of Place is required for the research mentioned in 1° of article L. 1121-1 of the Public Health Code, carried out outside places of care or in hospital departments when this research requires procedures other than those they usually carry out as part of their activity or when this research is carried out on people presenting a clinical condition distinct from that for which the department is responsible. The person in charge of the research is the principal investigator at each centre.

11.3.5 Informing the management and pharmacies of participating centres

The sponsor has ensured that the head of the institution and the pharmacist at the trial centre were informed before the start of the study and that an agreement was drawn up with each health institution participating in the protocol.

11.3.6 Informing subjects of overall research results

At the end of the study, if the volunteer so wishes, he or she may be informed of the overall results of the research (Article L1122-1, last paragraph). It is not possible for individuals to be informed of the individual results of the research, but they may be informed of their medical data. Patients must make their request in writing to the investigating doctor.

11.3.7 Audit and inspection

The investigators agree to comply with the requirements of the sponsor and the Competent Authority regarding an audit or inspection of the study.
The audit may apply to all stages of the study, from the development of the protocol to the publication of results and the classification of data used or produced as part of the study.

11.3.8 Archiving

At the end of the study, all documents relating to the study (including copies of observation notebooks) will be archived at the study site or in a centralised archive. Particular attention must be paid to the list identifying the patients included in the study and to the consent forms. This list and the consent forms are the most important documents in the files to be archived by the investigator.

All documents related to the study must be kept for 15 years after the end of the study. At the end of this period, the Sponsor will inform the investigators of the end of archiving.

12 FINANCING THE STUDY

This research project is funded by a DGOS PHRCI ***2019*** call for projects.

The budget will be managed by the Délégation à la Recherche Clinique et à l'Innovation in agreement with the investigator-coordinator of the study.

14. REPORTS AND PUBLICATIONS

13.1. Final report

The final study report will be written in collaboration with the study methodologists. It will be signed by the project coordinator and the sponsor and sent to each principal investigator. The results of the study, whatever they may be, will be submitted for publication.

13.2. Publication rules

The study will be reported to a register that meets the specifications required for publication of its results in major international medical journals, in accordance with the recommendations of the ICMJE (*International Committee of Medical Journal Editors*).
All data collected during this study are the property of the Study Sponsor and may not be disclosed to any third party under any circumstances without the written consent of the Study Co-ordinating Investigator.
The order of authors is defined as follows: each principal investigator of the centres and one associate investigator per centre will be included in the list of authors. The order of authors will be defined according to the number of patients included, and will ensure an equitable distribution of the ranks associated with the SIGAPS points. All co-investigators of the study will be grouped as co-authors within the OFACAR trial group.

Any publication or communication (oral or written) will be decided by mutual agreement between the investigators and will comply with the international recommendations of the ICMJE: Recommendations for the conduct, reporting, editing, and publication of scholarly work in medical journals. International Committee of Medical Journal Editors; Updated December 2016 www.icmje.org/icmje-recommendations.pdf.

For all publications, the investigator must refer to :

- at Dijon Bourgogne University Hospital
- the study funder (*contact DRCI to find out the terms required by the funder* (PHRC, Feder, Bourgogne region .....) - and if involved :
    - CRB Ferdinand Cabanne: In accordance with the standard published by the BRIF group (Bioresource Research Impact Factor), the identifier for the CRB Ferdinand Cabanne is BB0033-00044. This identifier should therefore be added to the CRB citation in the material.
      and methods: Biological Resource Center Ferdinand Cabanne BB-0033-00044 and mention this identifier in the references.
    - Inserm, CIC1432, module épidémiologie clinique ou plurithématique, F-21000, Dijon, France; for CIC units reporting to the CHU: CHU Dijon-Bourgogne, centre d'investigation clinique, module épidémiologie clinique/essais cliniques ou plurithématique, F-21000, Dijon, France.

In the case of publications produced jointly with the Université Bourgogne Franche Comté or any other research organisation, the rules for addressing publications will comply with the terms of the agreement, i.e. :

- Université Bourgogne Franche-Comté, *Name of laboratory and unit number*, F-21000 Dijon, France
- CHU Dijon Bourgogne, *Name of hospital department or laboratory*, F-21000 Dijon, France - Any other research organisation and/or establishment concerned by the publication

14. BIBLIOGRAPHY

1. Samii K, Fourcade O, Minville V, Geeraerts T. Traité d'Anesthésie-Réanimation. 2014.

2. Beloeil H, Laviolle B, Menard C, Paugam-Burtz C, Garot M, Asehnoune K, et al. POFA trial study protocol: a multicentre, double-blind, randomised, controlled clinical trial comparing opioid-free versus opioid anaesthesia on postoperative opioid-related adverse events after major or intermediate non-cardiac surgery. BMJ Open [Internet]. 30 June 2018 [cited 2 Sep 2019];8(6). Available from: https://www.ncbi.nlm.nih.gov/pmc/articles/PMC6042576/

3. Chen EY, Marcantonio A, Tornetta P. Correlation Between 24-Hour Predischarge Opioid Use and Amount of Opioids Prescribed at Hospital Discharge. JAMA Surg. Feb 1, 2018;153(2):e174859e174859.

4. Brummett CM, Waljee JF, Goesling J, Moser S, Lin P, Englesbe MJ, et al. New Persistent Opioid
Use After Minor and Major Surgical Procedures in US Adults. JAMA Surg. 2017 Jun 1;152(6):e170504-e170504.

5. Lee M, Silverman SM, Hansen H, Patel VB, Manchikanti L. A comprehensive review of opioidinduced hyperalgesia. Pain Physician. Apr 2011;14(2):145-61.

6. Jammer I, Wickboldt N, Sander M, Smith A, Schultz MJ, Pelosi P, et al. Standards for definitions and use of outcome measures for clinical effectiveness research in perioperative medicine: European Perioperative Clinical Outcome (EPCO) definitions: A statement from the ESA-ESICM joint taskforce on perioperative outcome measures. European Journal of Anaesthesiology (EJA). Feb 2015;32(2):88.

7. Ely EW, Margolin R, Francis J, May L, Truman B, Dittus R, et al. Evaluation of delirium in critically ill patients: Validation of the Confusion Assessment Method for the Intensive Care Unit (CAMICU). Critical Care Medicine. July 2001;29(7):1370.

8. Thielmann M, Sharma V, Al-Attar N, Bulluck H, Bisleri G, Bunge JJ, et al. ESC Joint Working Groups on Cardiovascular Surgery and the Cellular Biology of the Heart Position Paper: Perioperative myocardial injury and infarction in patients undergoing coronary artery bypass graft surgery. Eur Heart J. 14 August 2017;38(31):2392-411.

9. Kleif J, Waage J, Christensen KB, Gögenur I. Systematic review of the QoR-15 score, a patient- reported outcome measure measuring quality of recovery after surgery and anaesthesia. Br J Anaesth. Jan 2018;120(1):28-36.

10. Demumieux F, Ludes P-O, Diemunsch P, Bennett-Guerrero E, Lujic M, Lefebvre F, et al. Validation of the translated Quality of Recovery-15 questionnaire in a French-speaking population. British Journal of Anaesthesia. 1 June 2020;124(6):761-7.

11. Girard C, Mauriat Ph, Goudeau J-J, D'Athis P, Bompard D, Dalmas J-P, et al. L'anesthésie en chirurgie cardiaque adulte en France en 2001: Résultats de l'enquête nationale de trois jours du club Anesthésie-réanimation-techniques en chirurgie cardiaque (ARTECC). Annales Françaises d'Anesthésie et de Réanimation. 1 Sept 2004;23(9):862-72.

12. Saidman LJ, Bovill JG, Sebel PS, Stanley TH. Opioid Analgesics in AnesthesiaWith Special Reference to Their Use in Cardiovascular Anesthesia. Anesthes. 1984 Dec 1;61(6):731-55.

13. Howie MB, Smith DF, Reilley TE, McSweeney TD, Silver M, Dasta JF, et al. Postoperative course after sufentanil or fentanyl anesthesia for coronary artery surgery. Journal of Cardiothoracic and Vascular Anesthesia. 1 Oct 1991;5(5):485-9.

14. Miller DR, Wellwood M, Teasdale SJ, Laidley D, Ivanov J, Young P, et al. Effects of anaesthetic induction on myocardial function and metabolism: a comparison of fentanyl, sufentanil and alfentanil. Can J Anaesth. 1 May 1988;35(3):219-33.

15. Guinot P-G, Badoux L, Bernard E, Abou-Arab O, Lorne E, Dupont H. Central Venous-to-Arterial Carbon Dioxide Partial Pressure Difference in Patients Undergoing Cardiac Surgery is Not Related to Postoperative Outcomes. Journal of Cardiothoracic and Vascular Anesthesia. 1 August 2017;31(4):1190-6.

16. Forestier F, Hirschi M, Rouget P, Rigal J-C, Videcoq M, Girardet P, et al. Propofol and Sufentanil Titration with the Bispectral Index to Provide Anesthesia for Coronary Artery Surgery. Anesthes. 1 August 2003;99(2):334-46.

17. Jain U, Body SC, Bellows W, Wolman R, Mangano CM, Mathew J, et al. Multicenter Study of Target-Controlled Infusion of Propofol-Sufentanil or Sufentanil-Midazolam for Coronary Artery Bypass Graft Surgery. Anesthes. 1 Sep 1996;85(3):522-535.

18. Rivat C, Laboureyras E, Laulin J-P, Le Roy C, Richebé P, Simonnet G. Non-Nociceptive Environmental Stress Induces Hyperalgesia, Not Analgesia, in Pain and Opioid-Experienced Rats. Neuropsychopharmacology. Oct 2007;32(10):2217-28.

19. Célèrier E, Rivat C, Jun Y, Laulin J-P, Larcher A, Reynier P, et al. Long-lasting Hyperalgesia Induced by Fentanyl in Rats Preventive Effect of Ketamine. Anesthes. 1 Feb 2000;92(2):465-465.

20. Abrecht CR, Brovman EY, Greenberg P, Song E, Rathmell JP, Urman RD. A Contemporary Medicolegal Analysis of Outpatient Medication Management in Chronic Pain. Anesthesia & Analgesia. nov 2017;125(5):1761.

21. Pattinson KTS. Opioids and the control of respiration. British Journal of Anaesthesia. 1 June 2008;100(6):747-58.

22. Koo CY, Eikermann M. Respiratory Effects of Opioids in Perioperative Medicine. The Open Anesthesiology Journal [Internet]. 22 Jul 2011 [cited 2 Sep 2019];5(1). Available from: https://benthamopen.com/ABSTRACT/TOATJ-5-23

23. Steadman J, Catalani B, Sharp C, Cooper L. Life-threatening perioperative anaesthetic complications: major issues surrounding perioperative morbidity and mortality. Trauma Surg Acute Care Open. 1 Dec 2017;2(1):e000113.

24. Swart LM, van der Zanden V, Spies PE, de Rooij SE, van Munster BC. The Comparative Risk of Delirium with Different Opioids: A Systematic Review. Drugs Aging. 1 June 2017;34(6):437-43.

25. Burkhart CS, Dell-Kuster S, Gamberini M, Moeckli A, Grapow M, Filipovic M, et al. Modifiable and Nonmodifiable Risk Factors for Postoperative Delirium After Cardiac Surgery With Cardiopulmonary Bypass. Journal of Cardiothoracic and Vascular Anesthesia. August 1, 2010;24(4):555-9.

26. Dubowitz JA, Sloan EK, Riedel BJ. Implicating anaesthesia and the perioperative period in cancer recurrence and metastasis. Clin Exp Metastasis. Apr 1, 2018;35(4):347-58.

27. Stein C, Küchler S. Non-analgesic effects of opioids: peripheral opioid effects on inflammation and wound healing. Curr Pharm Des. 2012;18(37):6053-69.

28. Oderda GM, Evans RS, Lloyd J, Lipman A, Chen C, Ashburn M, et al. Cost of Opioid-Related Adverse Drug Events in Surgical Patients. Journal of Pain and Symptom Management. March 1, 2003;25(3):276-83.

29. Laborit H. L'Anesthésié Facilitée par les Synergies Medicamenteuses. Anesthesia & Analgesia. Oct 1951;30(5):284.

30. Mulier JP. Why and how to give opioid free anaesthesia (OFA). The Bruges approach. American Society of anesthesiologists Annual meeting, New Orleans; 2016.

31. Mulier J, Wouters R, Dillemans B, Dekock M. A Randomized Controlled, Double-Blind Trial Evaluating the Effect of Opioid-Free Versus Opioid General Anaesthesia on Postoperative Pain and Discomfort Measured by the QoR-40. 27 Feb 2018;2.

32. Bakan M, Umutoglu T, Topuz U, Uysal H, Bayram M, Kadioglu H, et al. Opioid-free total intravenous anaesthesia with propofol, dexmedetomidine and lidocaine infusions for laparoscopic cholecystectomy: a prospective, randomized, double-blinded study. Brazilian Journal of Anesthesiology (English Edition). 1 May 2015;65(3):191-9.

33. Tripathy S, Rath S, Agrawal S, Rao PB, Panda A, Mishra TS, et al. Opioid-free anaesthesia for breast cancer surgery: An observational study. J Anaesthesiol Clin Pharmacol. 2018;34(1):35-40.

34. Ziemann-Gimmel P, Goldfarb AA, Koppman J, Marema RT. Opioid-free total intravenous anaesthesia reduces postoperative nausea and vomiting in bariatric surgery beyond triple prophylaxis. British Journal of Anaesthesia. 1 May 2014;112(5):906-11.

35. Coloma M, Chiu JW, White PF, Armbruster SC. The Use of Esmolol as an Alternative to Remifentanil During Desflurane Anesthesia for Fast-Track Outpatient Gynecologic Laparoscopic Surgery. Anesthesia & Analgesia. Feb 2001;92(2):352.

36. De Windt AC, Asehnoune K, Roquilly A, Guillaud C, Le Roux C, Pinaud M, et al. An opioid-free anaesthetic using nerve blocks enhances rapid recovery after minor hand surgery in children. European Journal of Anaesthesiology (EJA). June 2010;27(6):521.

37. Callesen T, Schouenborg L, Nielsen D, Guldager H, Kehlet H. Combined epidural-spinal opioidfree anaesthesia and analgesia for hysterectomy. Br J Anaesth. June 1999;82(6):881-5.

38. Brandal D, Keller MS, Lee C, Grogan T, Fujimoto Y, Gricourt Y, et al. Impact of Enhanced Recovery After Surgery and Opioid-Free Anesthesia on Opioid Prescriptions at Discharge From the Hospital: A Historical-Prospective Study. Anesthesia & Analgesia. nov 2017;125(5):1784.

39. Gaszynski T. Opioid-free general anesthesia in patient with Steinert syndrome (myotonic dystrophy). Medicine (Baltimore) [Internet]. 2016 Sep 16 [cited 2019 Sep 2];95(37). Available from: https://www.ncbi.nlm.nih.gov/pmc/articles/PMC5402602/

40. Naik BI, Tsang S, Knisely A, Yerra S, Durieux ME. Retrospective case-control non-inferiority analysis of intravenous lidocaine in a colorectal surgery enhanced recovery program. BMC Anesthesiol [Internet]. 31 Jan 2017 [cited 2 Sep 2019];17. Available from: https://www.ncbi.nlm.nih.gov/pmc/articles/PMC5282801/

41. Plunkett A, Fahlgren M, McLean B, Mundey D. Opioid-Free Balanced Anesthesia for Cervical Ganglionectomy Subsequent to Recent Ultra Rapid Opioid Detoxification. Pain Med. 1 May 2009;10(4):767-70.

42. Waldron NH, Jones CA, Gan TJ, Allen TK, Habib AS. Impact of perioperative dexamethasone on postoperative analgesia and side-effects: systematic review and meta-analysis. British Journal of Anaesthesia. 1 Feb 2013;110(2):191-200.

43. Harless M, Depp C, Collins SB, Hewer I. Role of Esmolol in Perioperative Analgesia and Anesthesia: A Literature Review. AANA journal. 2015;83(3):167-77.

44. Gelineau AM, King MR, Ladha KS, Burns SM, Houle T, Anderson TA. Intraoperative Esmolol as an Adjunct for Perioperative Opioid and Postoperative Pain Reduction: A Systematic Review, Meta-analysis, and Meta-regression. Anesthesia & Analgesia. march 2018;126(3):1035.

45. Collard V, Mistraletti G, Taqi A, Asenjo JF, Feldman LS, Fried GM, et al. Intraoperative Esmolol Infusion in the Absence of Opioids Spares Postoperative Fentanyl in Patients Undergoing Ambulatory Laparoscopic Cholecystectomy. Anesthesia & Analgesia. Nov 2007;105(5):1255.

46. Asouhidou I, Trikoupi A. Esmolol reduces anaesthetic requirements thereby facilitating early extubation; a prospective controlled study in patients undergoing intracranial surgery. BMC Anesthesiol [Internet]. 28 Nov 2015 [cited 2 Sep 2019];15. Available from: https://www.ncbi.nlm.nih.gov/pmc/articles/PMC4663038/

47. Çakırgöz MY, Taşdöğen A, Olguner CG, Korkmaz HT, Oğün E, Küçükebe B, et al. The effect of different doses of esmolol on hemodynamic, bispectral index and movement response during orotracheal intubation: prospective, randomized, double-blind study. Revista brasileira de anestesiologia. 2014;64(6):425-32.

48. Becker DE, Reed KL. Local Anesthetics: Review of Pharmacological Considerations. Anesth Prog. 2012;59(2):90-102.

49. Groudine SB, Fisher HAG, Kaufman RP, Patel MK, Wilkins LJ, Mehta SA, et al. Intravenous Lidocaine Speeds the Return of Bowel Function, Decreases Postoperative Pain, and Shortens Hospital Stay in Patients Undergoing Radical Retropubic Prostatectomy. Anesthesia & Analgesia. Feb 1998;86(2):235.

50. Hollmann MW, Durieux ME. Local Anesthetics and the Inflammatory ResponseA New Therapeutic Indication? Anesthes. 1 Sep 2000;93(3):858-75.

51. Koppert W, Weigand M, Neumann F, Sittl R, Schuettler J, Schmelz M, et al. Perioperative Intravenous Lidocaine Has Preventive Effects on Postoperative Pain and Morphine Consumption After Major Abdominal Surgery. Anesthesia & Analgesia. Apr 2004;98(4):1050.

52. De Oliveira GS, Fitzgerald P, Streicher LF, Marcus R-J, McCarthy RJ. Systemic Lidocaine to Improve Postoperative Quality of Recovery After Ambulatory Laparoscopic Surgery. Anesthesia & Analgesia. August 2012;115(2):262.

53. Herroeder S, Pecher S, Schönherr ME, Kaulitz G, Hahnenkamp K, Friess H, et al. Systemic
Lidocaine Shortens Length of Hospital Stay After Colorectal Surgery. Ann Surg. August 2007;246(2):192-200.

54. Baradari AG, Habibi MR, Habibi V, Nouraei SM. Administration of lidocaine to prevent cognitive deficit in patients undergoing coronary artery bypass grafting and valve plasty: a systematic review and meta-analysis. Expert Review of Clinical Pharmacology. Feb 1, 2017;10(2):179-85.
55. Dobson GP. Organ arrest, protection and preservation: natural hibernation to cardiac surgery. Comparative Biochemistry and Physiology Part B: Biochemistry and Molecular Biology. 1 Nov 2004;139(3):469-85.

56. Weibel S, Jelting Y, Pace NL, Helf A, Eberhart LH, Hahnenkamp K, et al. Continuous intravenous perioperative lidocaine infusion for postoperative pain and recovery in adults. Cochrane Database Syst Rev [Internet]. 2018 Jun 4 [cited 2019 Sep 2];2018(6). Available from: https://www.ncbi.nlm.nih.gov/pmc/articles/PMC6513586/

57. Ghafari R, Baradari AG, Firouzian A, Nouraei M, Aarabi M, Zamani A, et al. Cognitive deficit in first-time coronary artery bypass graft patients: a randomized clinical trial of lidocaine versus procaine hydrochloride. Perfusion. 1 Jul 2012;27(4):320-5.

58. Klinger RY, Cooter M, Berger M, Podgoreanu MV, Stafford-Smith M, Ortel TL, et al. Effect of intravenous lidocaine on the transcerebral inflammatory response during cardiac surgery: a randomized-controlled trial. Can J Anesth/J Can Anesth. 1 Nov 2016;63(11):1223-32.

59. Fiore AC, Naunheim KS, Taub J, Braun P, McBride LR, Pennington DG, et al. Myocardial preservation using lidocaine blood cardioplegia. Ann Thorac Surg. Nov 1990;50(5):771-5.

60. Benowitz NL, Meister W. Clinical pharmacokinetics of lignocaine. Clin Pharmacokinet. June 1978;3(3):177-201.

61. Jonkman K, Dahan A, van de Donk T, Aarts L, Niesters M, van Velzen M. Ketamine for pain. F1000Res [Internet]. 20 Sep 2017 [cited 2 Sep 2019];6. Available from:
https://www.ncbi.nlm.nih.gov/pmc/articles/PMC5609085/

62. Jouguelet-Lacoste J, La Colla L, Schilling D, Chelly JE. The Use of Intravenous Infusion or Single Dose of Low-Dose Ketamine for Postoperative Analgesia: A Review of the Current Literature. Pain Med. Feb 1, 2015;16(2):383-403.

63. Hudetz JA, Iqbal Z, Gandhi SD, Patterson KM, Byrne AJ, Hudetz AG, et al. Ketamine attenuates post-operative cognitive dysfunction after cardiac surgery. Acta Anaesthesiologica Scandinavica. 2009;53(7):864-72.

64. Chassot P. Specificity of anaesthesia for cardiac surgery [Internet]. Available at: http://www.pac4.ch/chapitre4/Remarqgener.html

65. Cook DJ, Rooke GA. Priorities in Perioperative Geriatrics. Anesthesia & Analgesia. June 2003;96(6):1823.

66. Fischer M-O. Hemodynamic Monitoring, Positive Inotropic and Vasoactive Drugs During Cardiac Surgery (EMOA) - NCT02571920 [Internet]. [cited 2 Sep 2019]. Available from:
https://clinicaltrials.gov/ct2/show/NCT02571920

67. Jokela RM, Ahonen JV, Tallgren MK, Marjakangas PC, Korttila KT. The Effective Analgesic Dose of Dexamethasone After Laparoscopic Hysterectomy. Anesthesia & Analgesia. August 2009;109(2):607.

68. Guinot P-G, Spitz A, Berthoud V, Ellouze O, Missaoui A, Constandache T, et al. Effect of opioid-free anaesthesia on post-operative period in cardiac surgery: a retrospective matched case-control study. BMC Anesthesiol

[Internet]. 31 Jul 2019 [cited 2 Sep 2019];19. Available from: https://www.ncbi.nlm.nih.gov/pmc/articles/PMC6668113/

69. Bern S, Akpa BS, Kuo I, Weinberg G. Lipid resuscitation: a life-saving antidote for local anaesthetic toxicity. Curr Pharm Biotechnol. 1 Feb 2011;12(2):313-9.

70. Acute Respiratory Distress Syndrome: The Berlin Definition. JAMA. 20 June 2012;307(23):252633.

71. Khwaja A. KDIGO Clinical Practice Guidelines for Acute Kidney Injury. NEC. 2012;120(4):c17984.

72. Dyke C, Aronson S, Dietrich W, Hofmann A, Karkouti K, Levi M, et al. Universal definition of perioperative bleeding in adult cardiac surgery. The Journal of Thoracic and Cardiovascular Surgery. 1 May 2014;147(5):1458-1463.e1.

73. Shankar-Hari M, Phillips GS, Levy ML, Seymour CW, Liu VX, Deutschman CS, et al. Developing a New Definition and Assessing New Clinical Criteria for Septic Shock. JAMA. Feb 23, 2016;315(8):775-87.
